# Supplementary material for: Immune aging impairs muscle regeneration via macrophage-derived anti-oxidant selenoprotein P
Source: EMBO Rep. 2025 Jul 18;26(16):4153–79. doi: 10.1038/s44319-025-00516-3 (PMC12373998; doi:10.1038/s44319-025-00516-3)
Supplement: Supplementary file 1 — Appendix [file 44319_2025_516_MOESM1_ESM.pdf]

Immune aging impairs muscle regeneration via macrophage-derived anti-oxidant selenoprotein P

Appendix

Content:

Appendix Figure S1	page 2
Appendix Figure S2	page 3

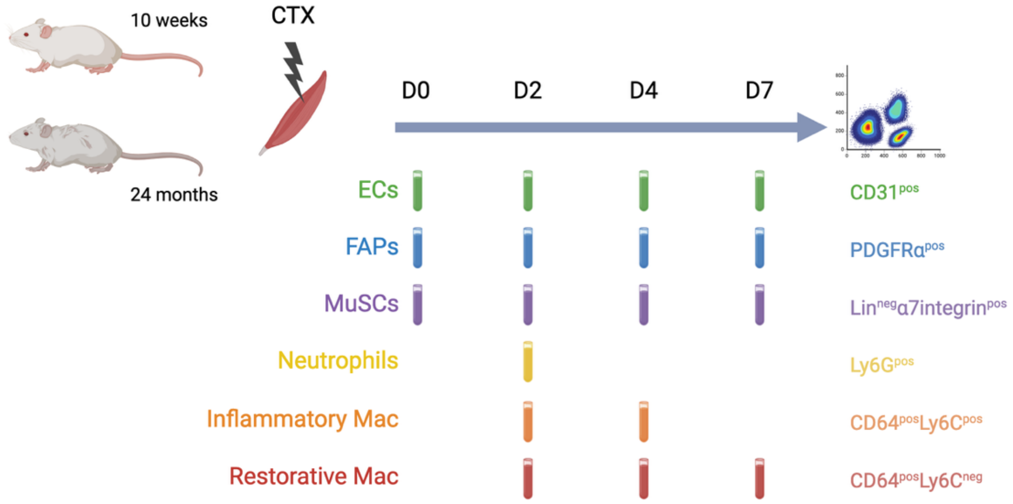

Appendix Figure S1. Experimental design for isolation of cells from young and aged regenerating muscle. MuSCs, ECs, FAPs, Neutrophils, Inflammatory macrophages and restorative macrophages were FACs isolated from young and old muscle before and at days 2, 4 and 7 after the injury and were processed for bulk-RNASeq.

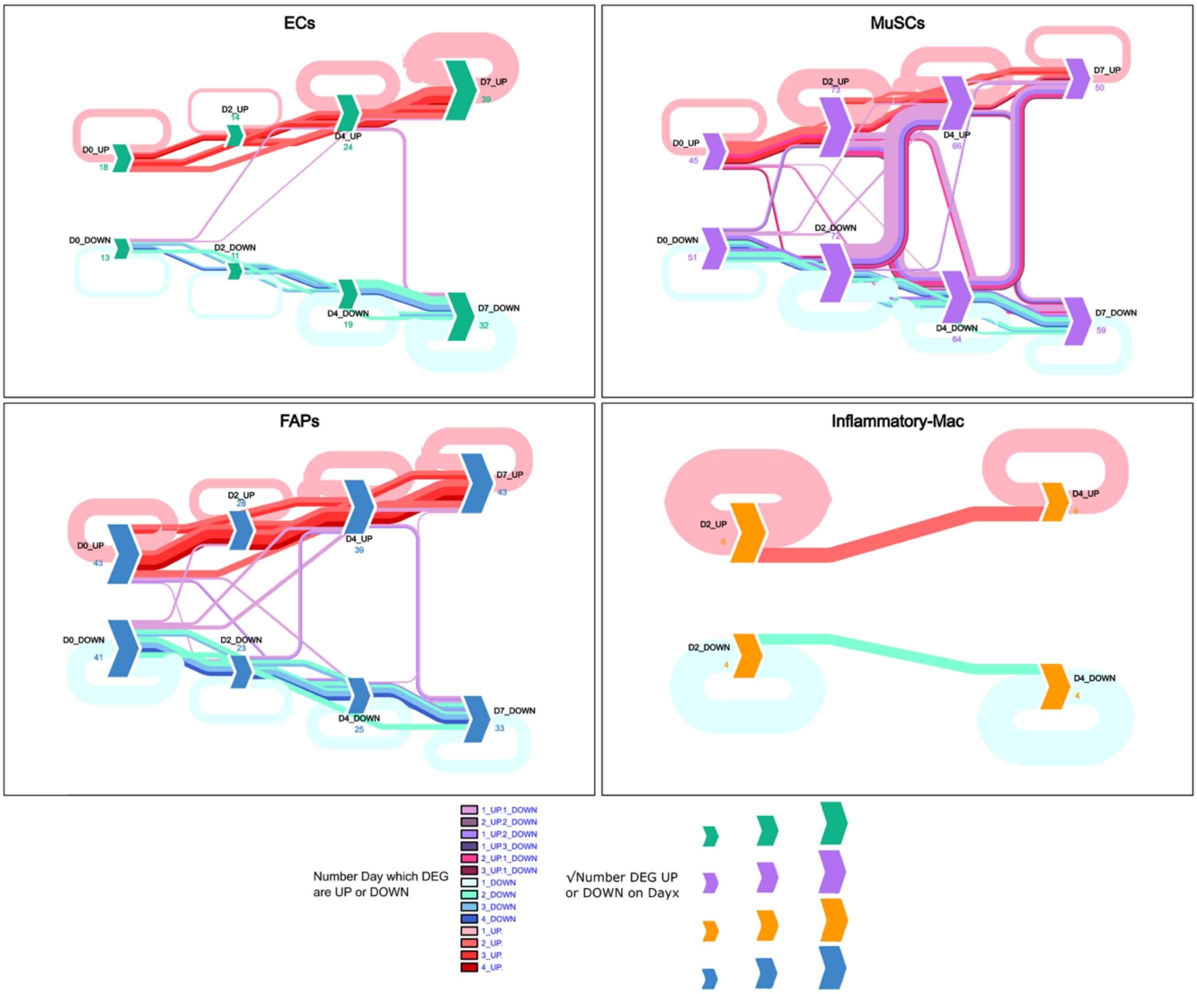

Appendix Figure S2. Differentially expressed genes (DEG) in old *versus* young mononucleated cells. Differentially expressed gene cycle and flow in cells during regeneration with segregation of upregulated genes (reddish colors) and downregulated (blueish colors). Note the purple flows showing genes that change their regulation during regeneration.
