# Supplementary figures and images for: Immune aging impairs muscle regeneration via macrophage-derived anti-oxidant selenoprotein P
Source: EMBO Rep. 2025 Jul 18;26(16):4153–79. doi: 10.1038/s44319-025-00516-3 (PMC12373998; doi:10.1038/s44319-025-00516-3)

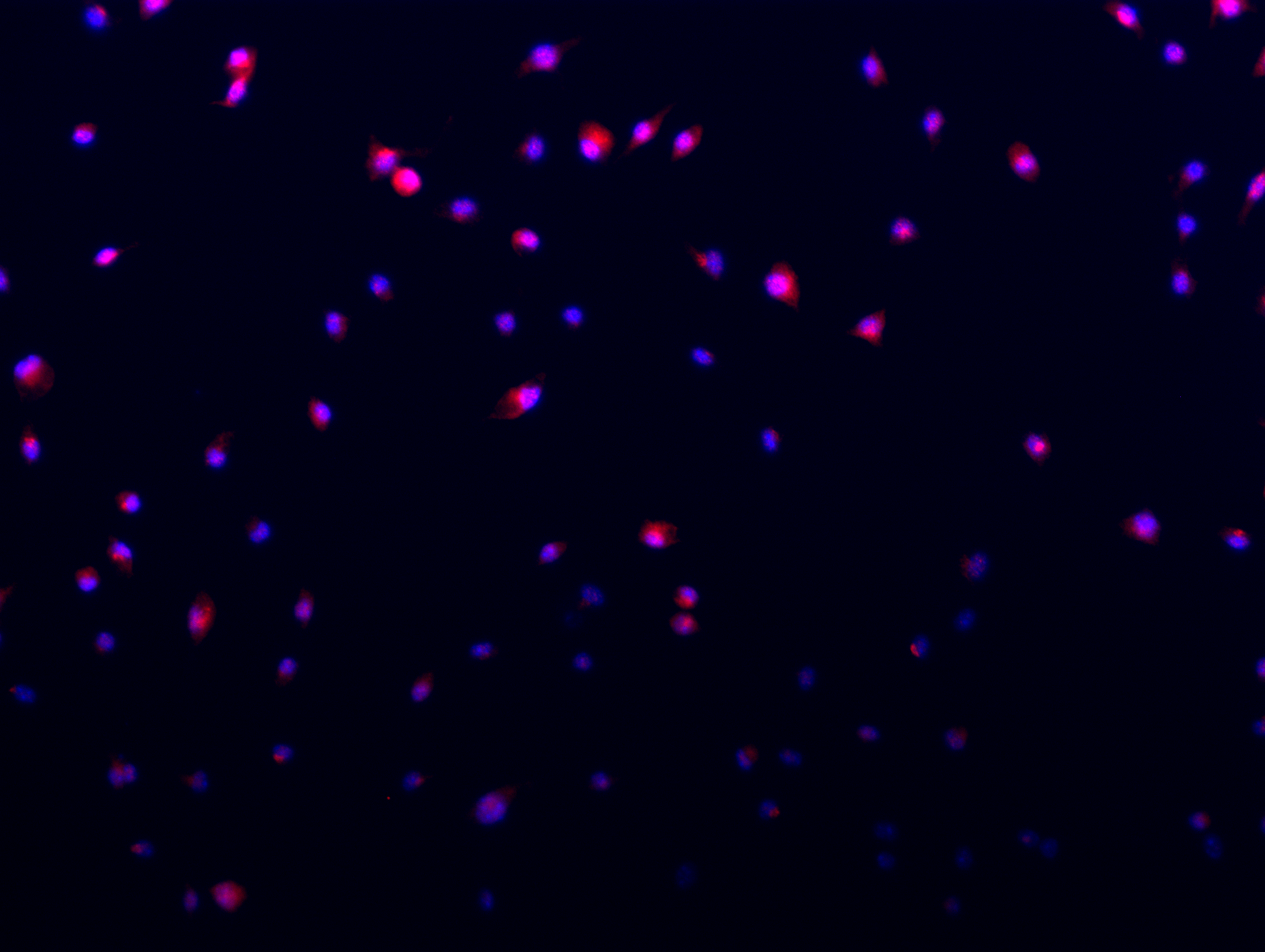

Supplement: Supplementary file 4 — Source data Fig. 4 [file 44319_2025_516_MOESM4_ESM.zip › Figure 4/4B/IF iNOS Sepp1KO IFNg.tif (RGB).tif]

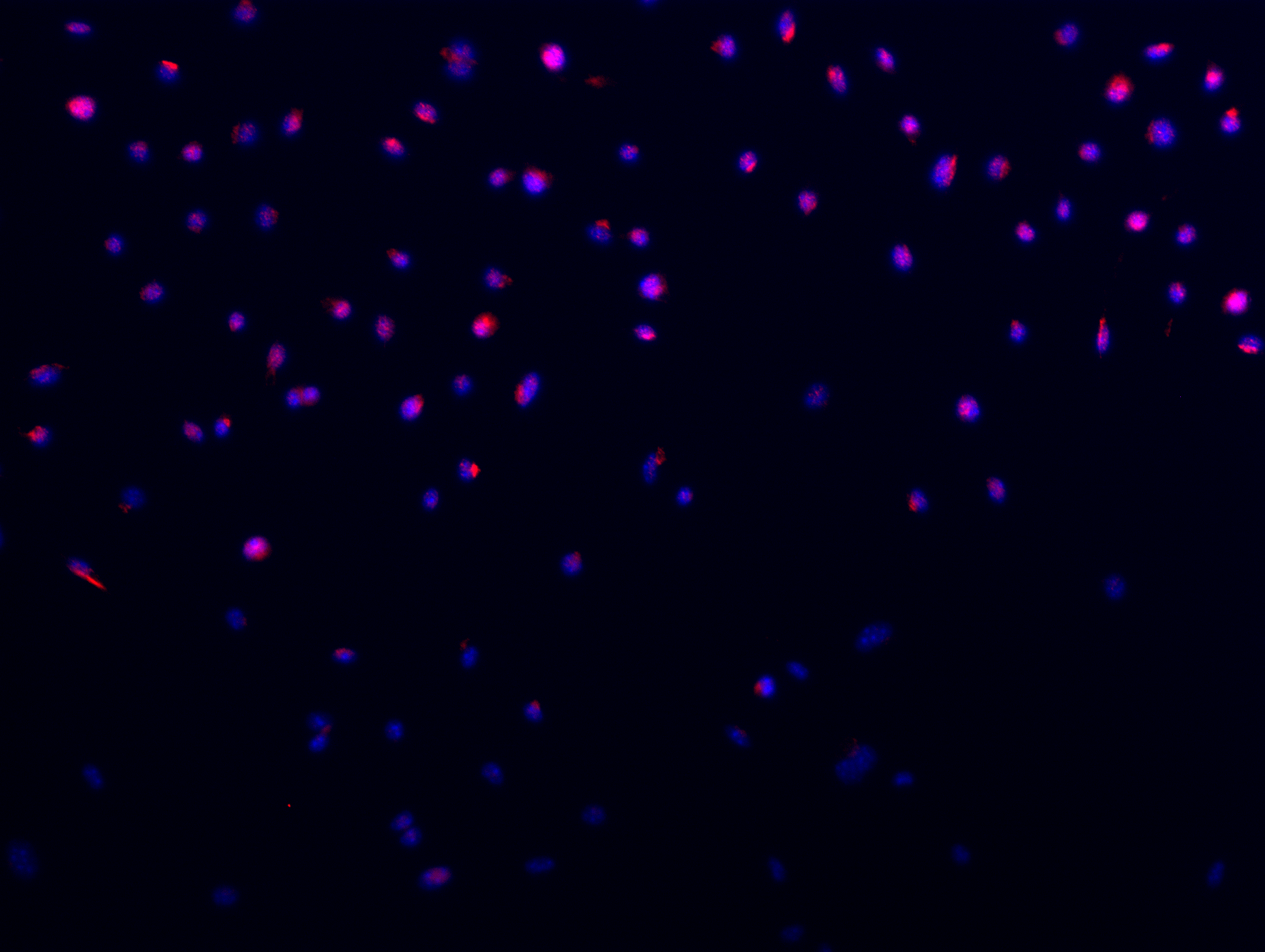

Supplement: Supplementary file 4 — Source data Fig. 4 [file 44319_2025_516_MOESM4_ESM.zip › Figure 4/4B/IF iNOS Sepp1KO IL10.tif (RGB).tif]

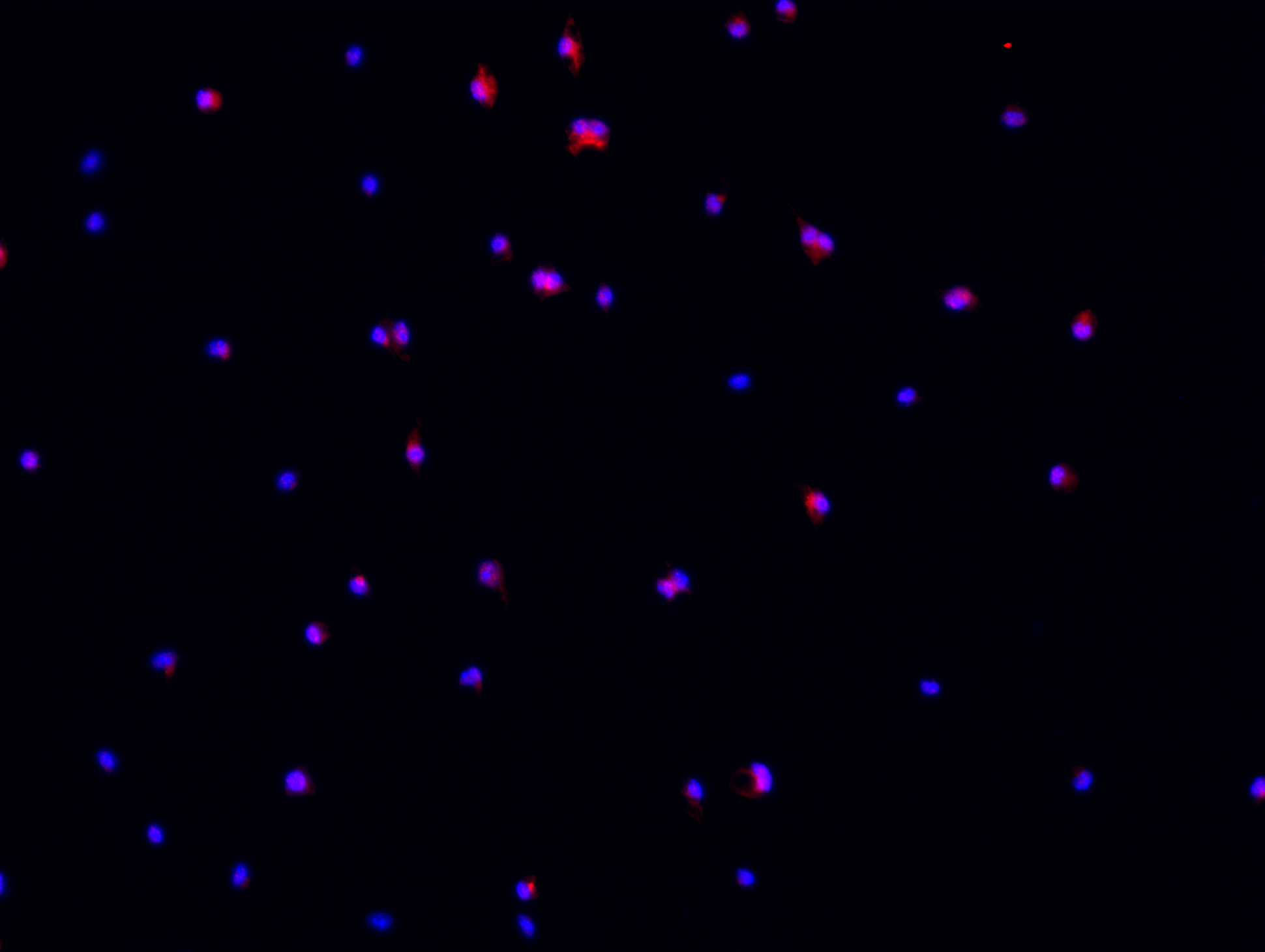

Supplement: Supplementary file 4 — Source data Fig. 4 [file 44319_2025_516_MOESM4_ESM.zip › Figure 4/4B/IF iNOS WT IFNg.tif (RGB).tif]

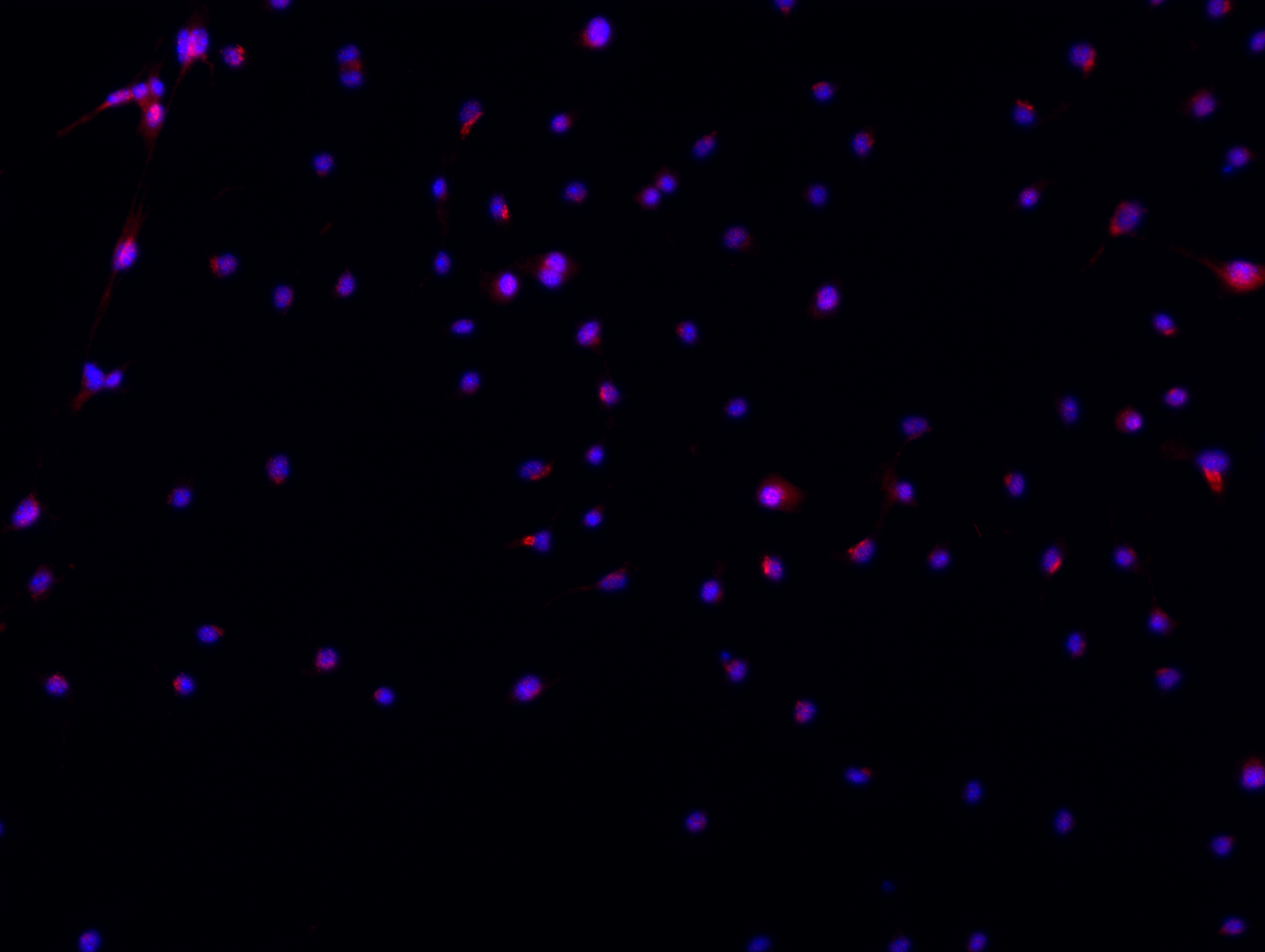

Supplement: Supplementary file 4 — Source data Fig. 4 [file 44319_2025_516_MOESM4_ESM.zip › Figure 4/4B/IF iNOS WT IL10.tif (RGB).tif]

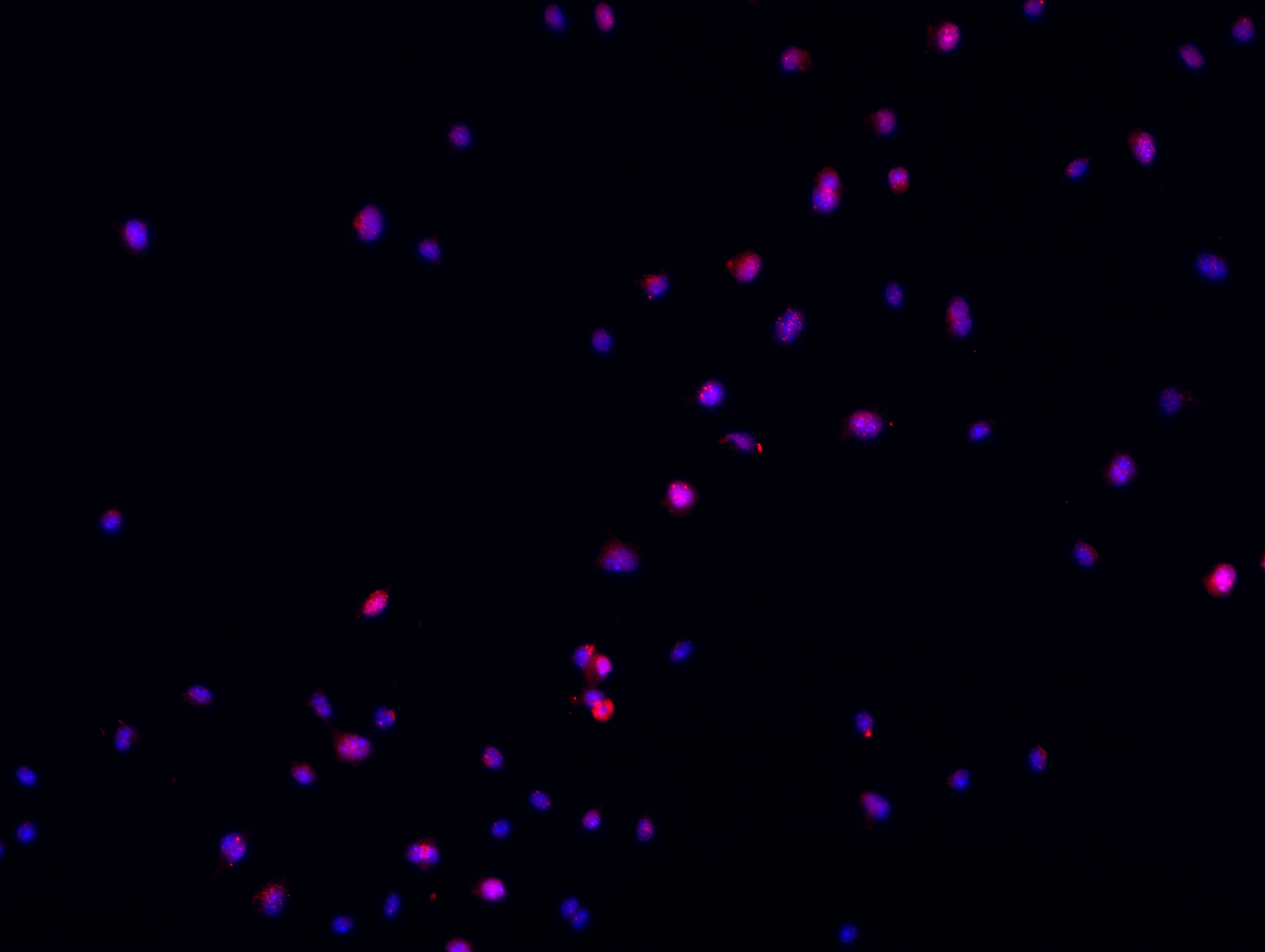

Supplement: Supplementary file 4 — Source data Fig. 4 [file 44319_2025_516_MOESM4_ESM.zip › Figure 4/4C/IF CCL3 Sepp1KO IFNg.tif (RGB).tif]

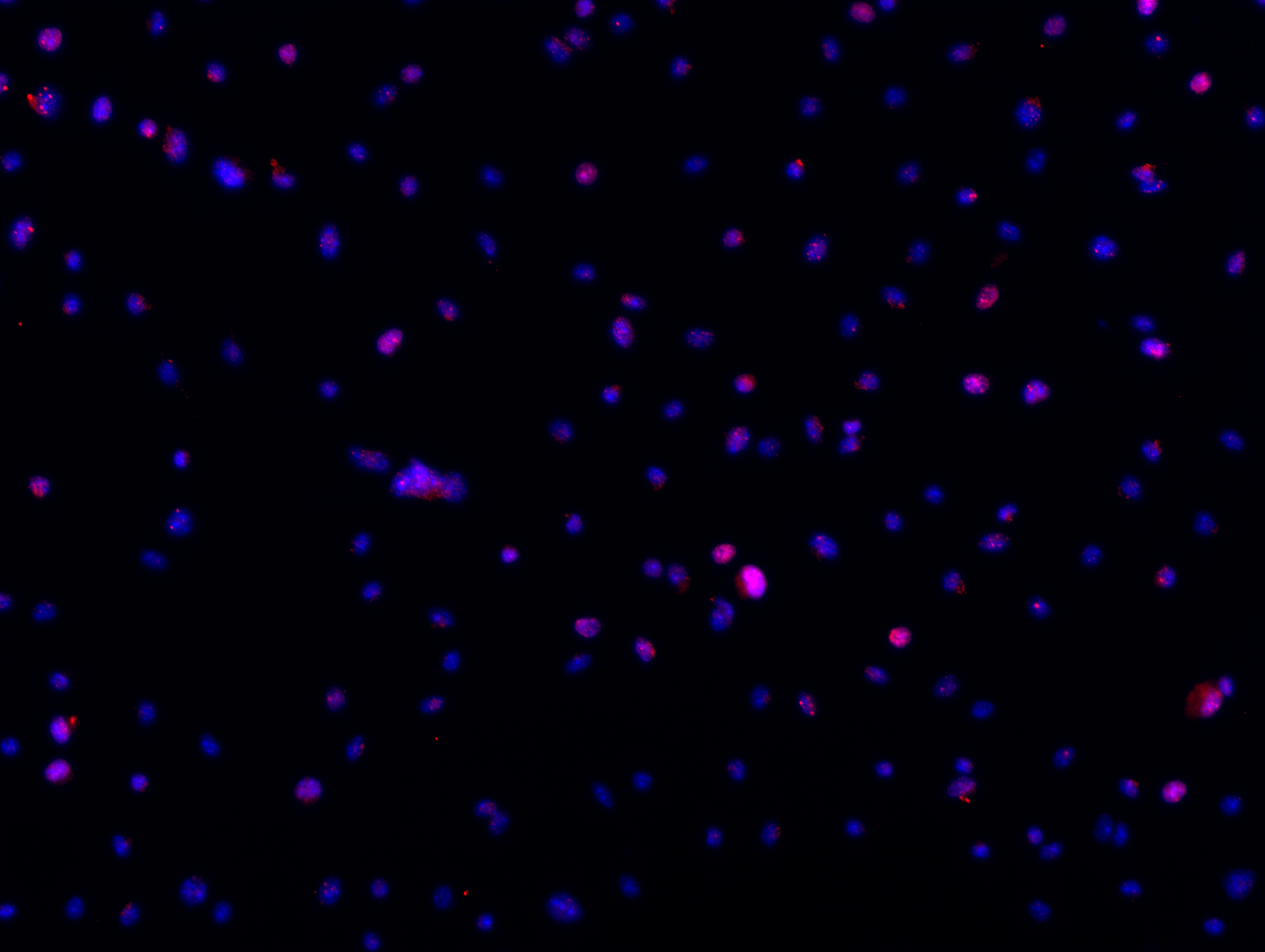

Supplement: Supplementary file 4 — Source data Fig. 4 [file 44319_2025_516_MOESM4_ESM.zip › Figure 4/4C/IF CCL3 Sepp1KO IL10.tif (RGB).tif]

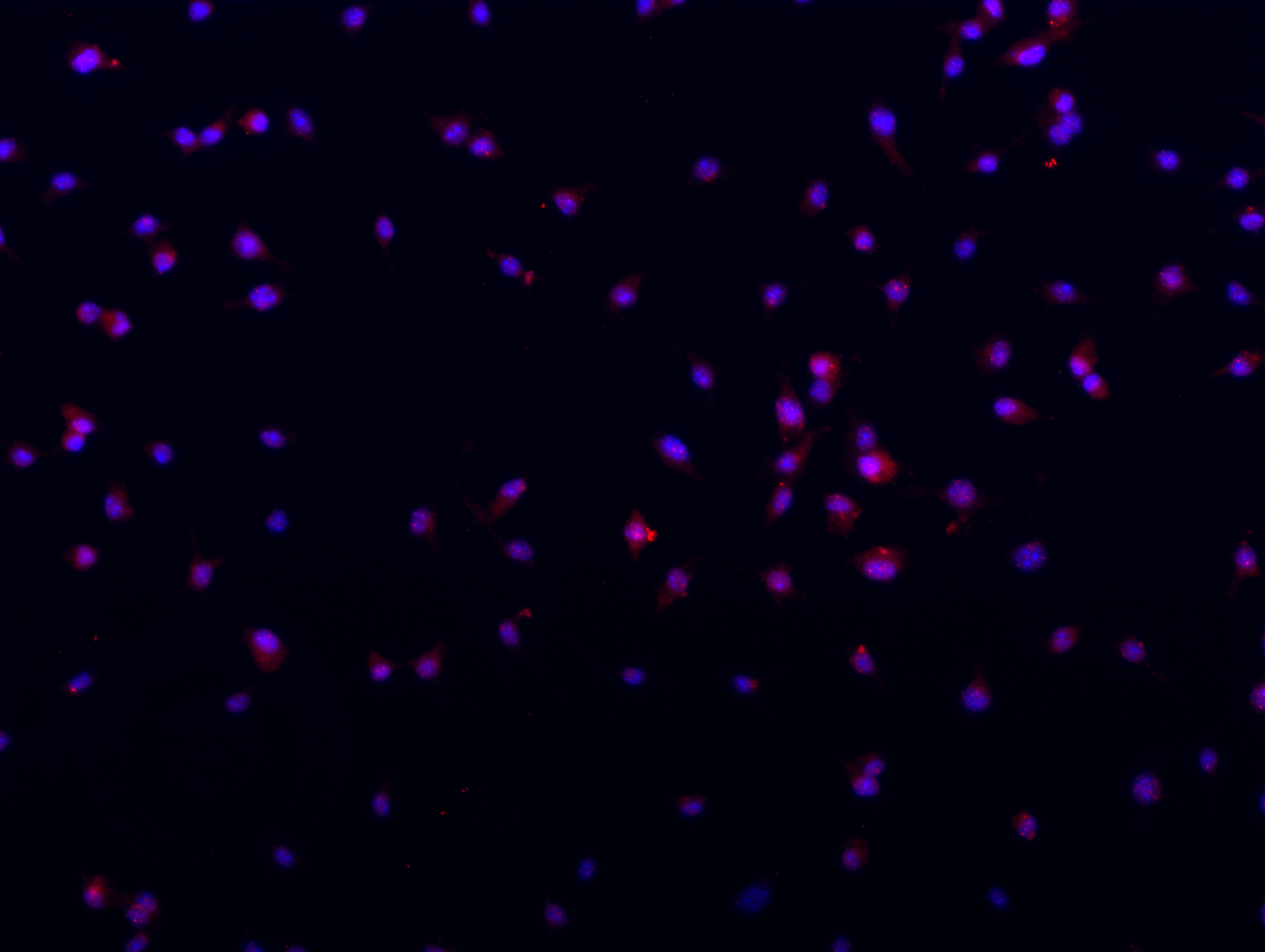

Supplement: Supplementary file 4 — Source data Fig. 4 [file 44319_2025_516_MOESM4_ESM.zip › Figure 4/4C/IF CCL3 WT IFNg.tif (RGB).tif]

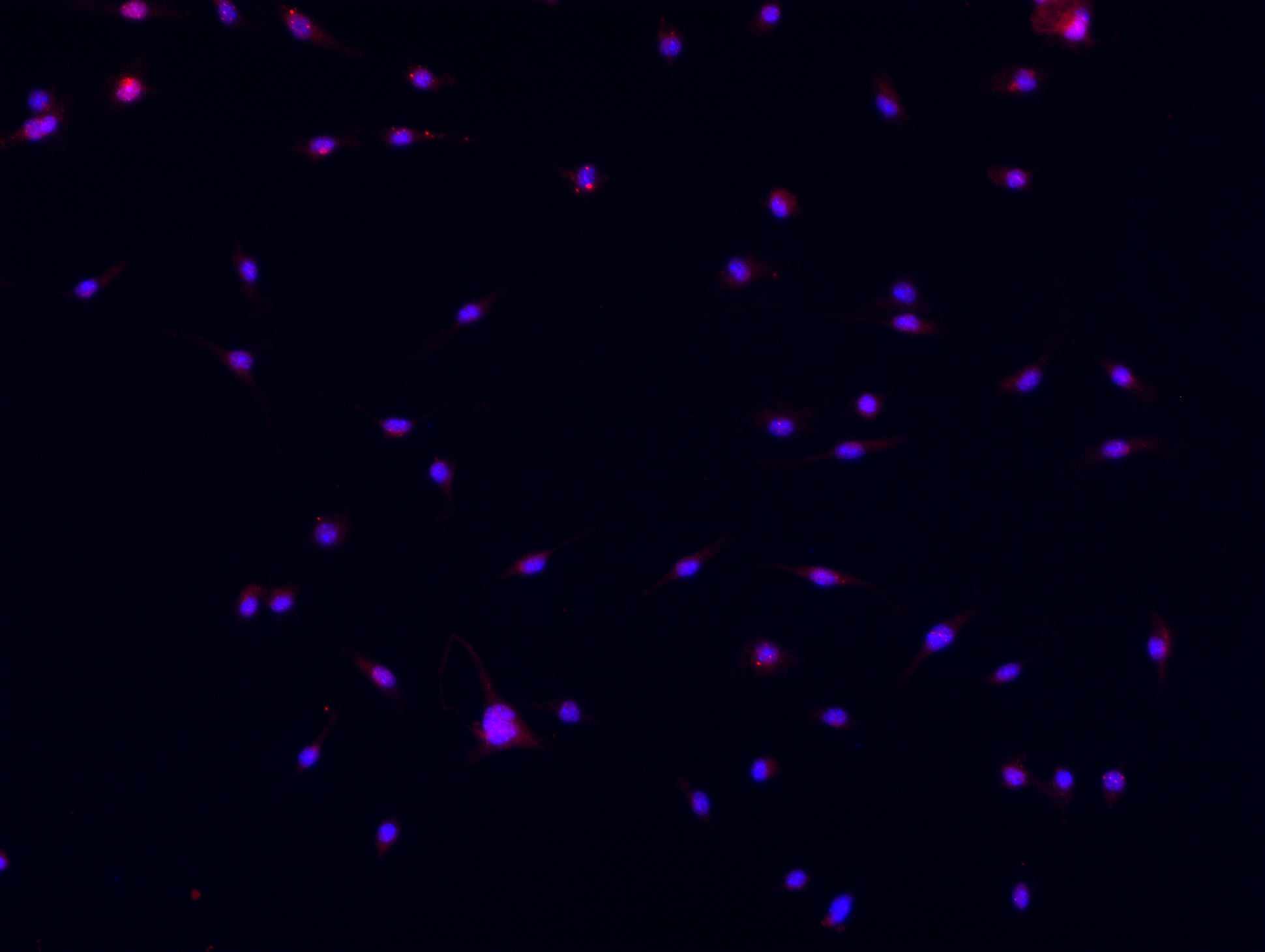

Supplement: Supplementary file 4 — Source data Fig. 4 [file 44319_2025_516_MOESM4_ESM.zip › Figure 4/4C/IF CCL3 WT IL10.tif (RGB).tif]

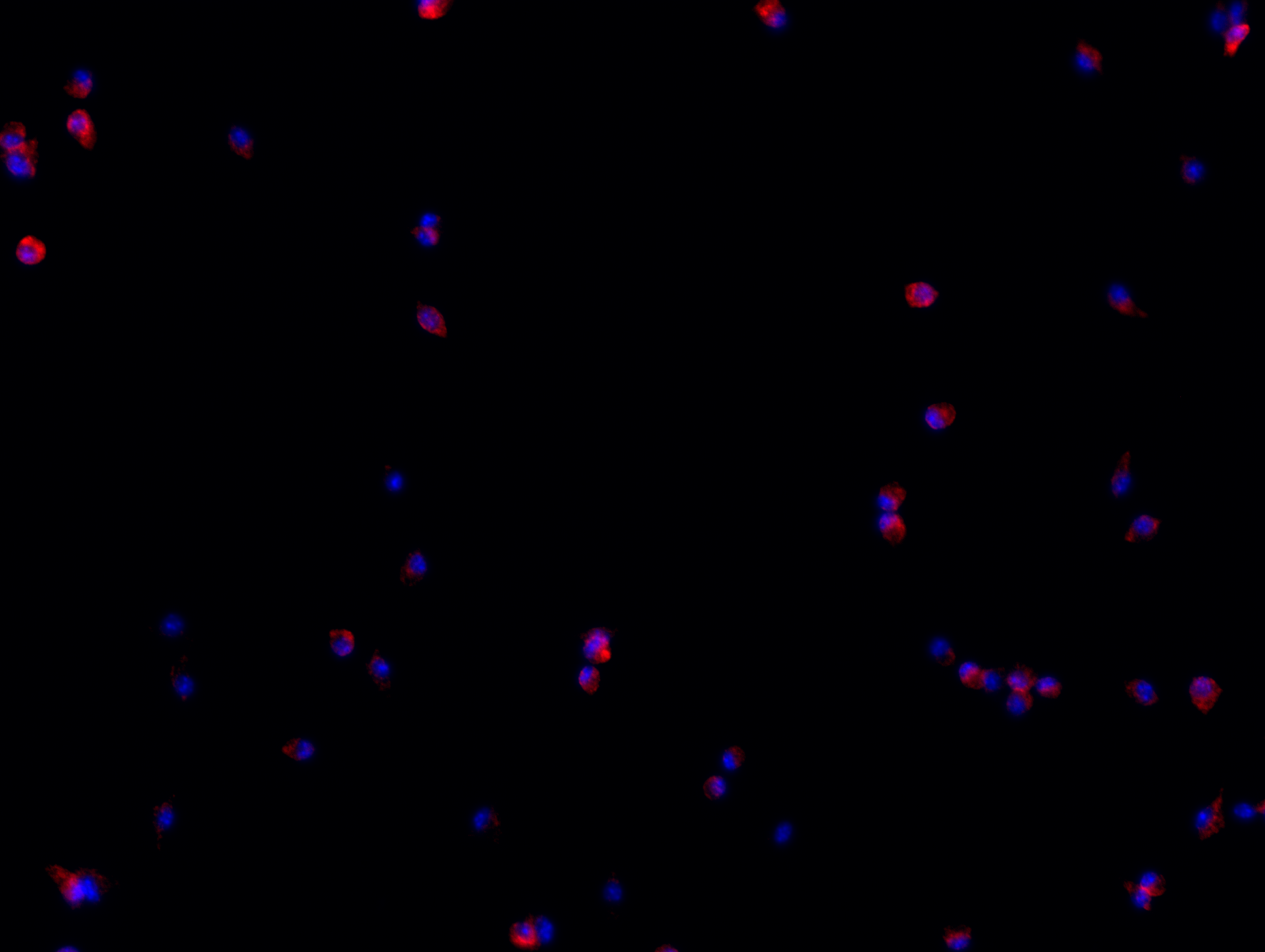

Supplement: Supplementary file 4 — Source data Fig. 4 [file 44319_2025_516_MOESM4_ESM.zip › Figure 4/4D/IF CD206 Sepp1KO IFNg.tif (RGB).tif]

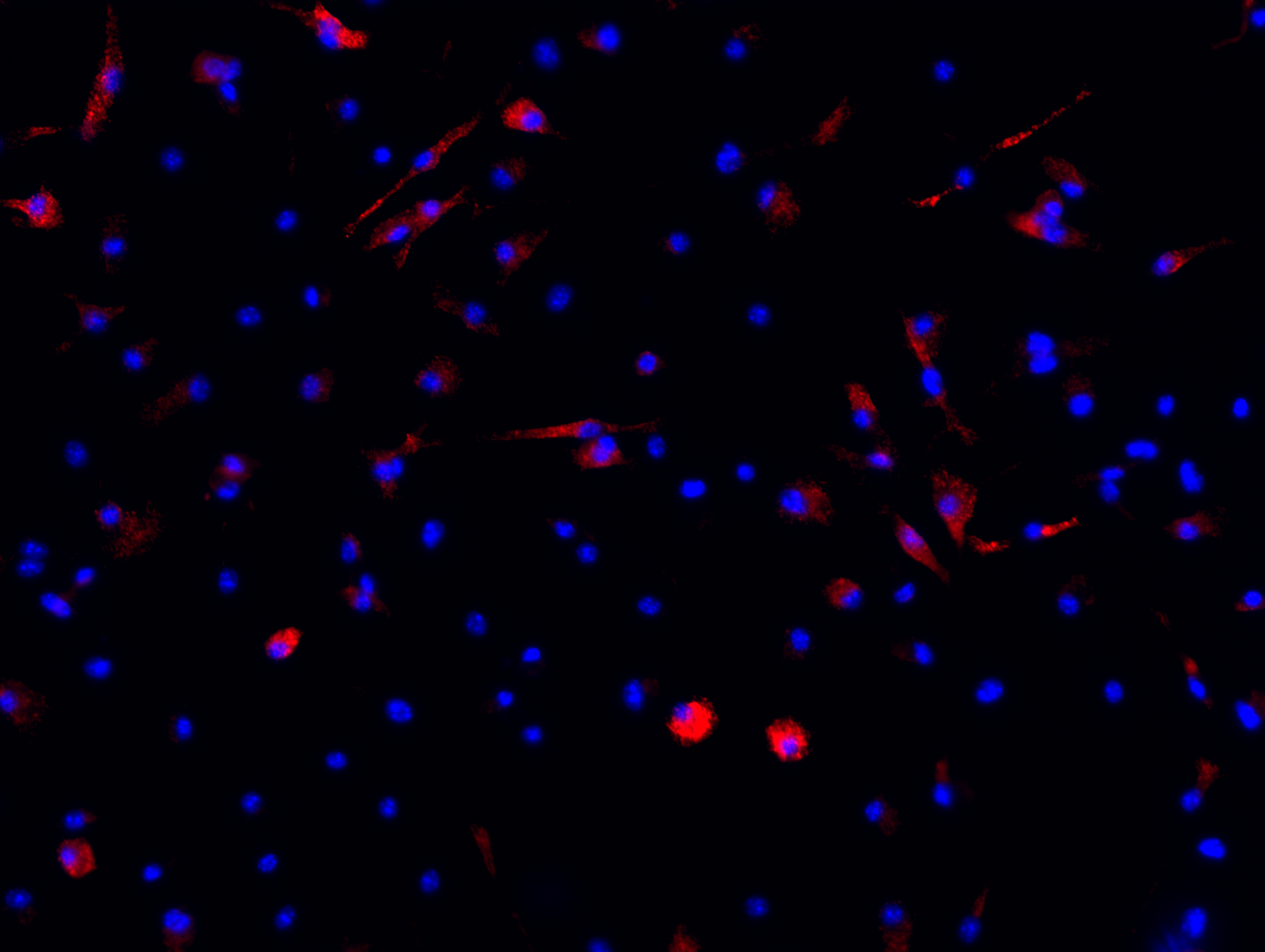

Supplement: Supplementary file 4 — Source data Fig. 4 [file 44319_2025_516_MOESM4_ESM.zip › Figure 4/4D/IF CD206 Sepp1KO IL10.tif (RGB).tif]

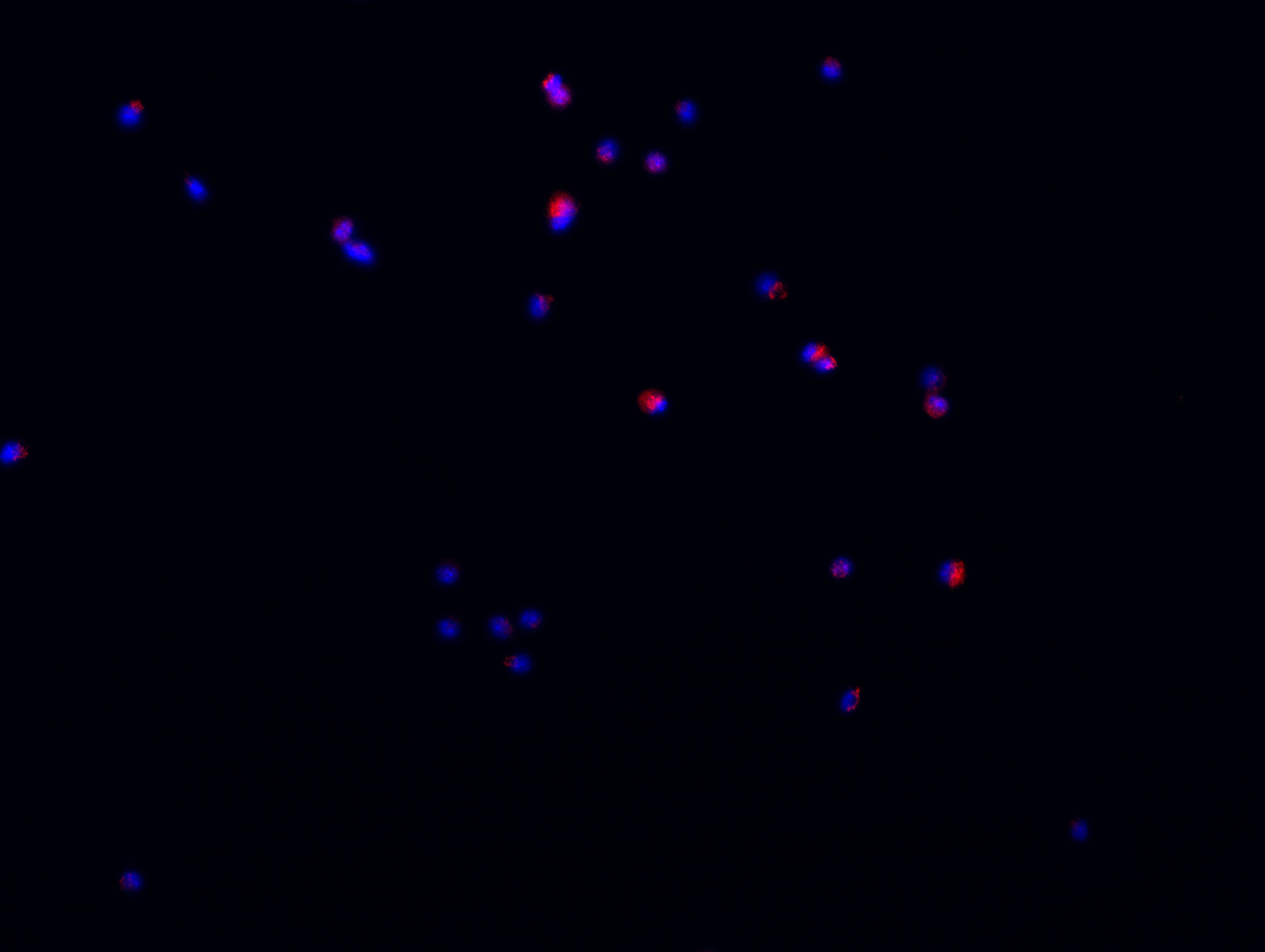

Supplement: Supplementary file 4 — Source data Fig. 4 [file 44319_2025_516_MOESM4_ESM.zip › Figure 4/4D/IF CD206 WT IFNg.tif (RGB).tif]

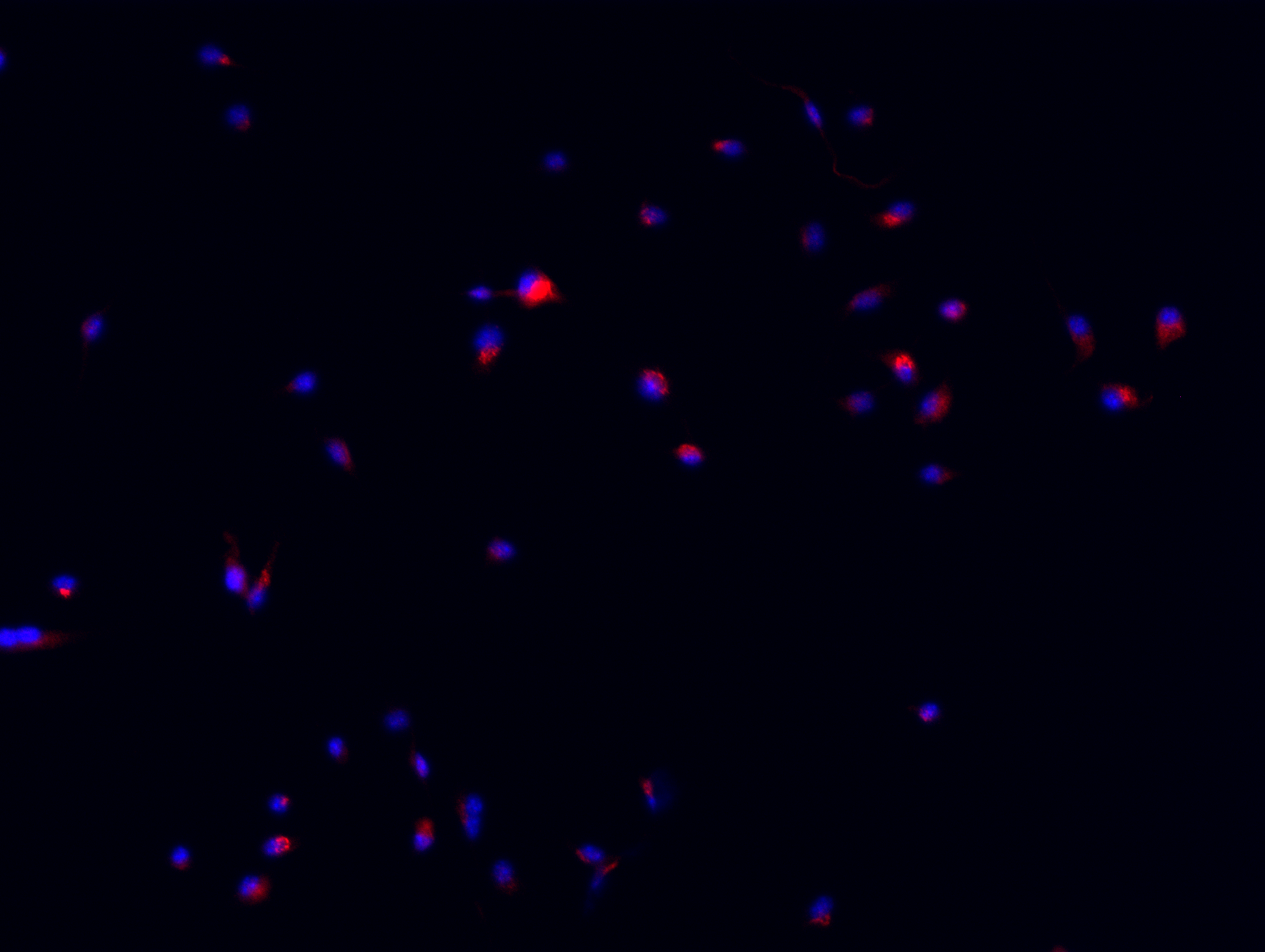

Supplement: Supplementary file 4 — Source data Fig. 4 [file 44319_2025_516_MOESM4_ESM.zip › Figure 4/4D/IF CD206 WT IL10.tif (RGB).tif]

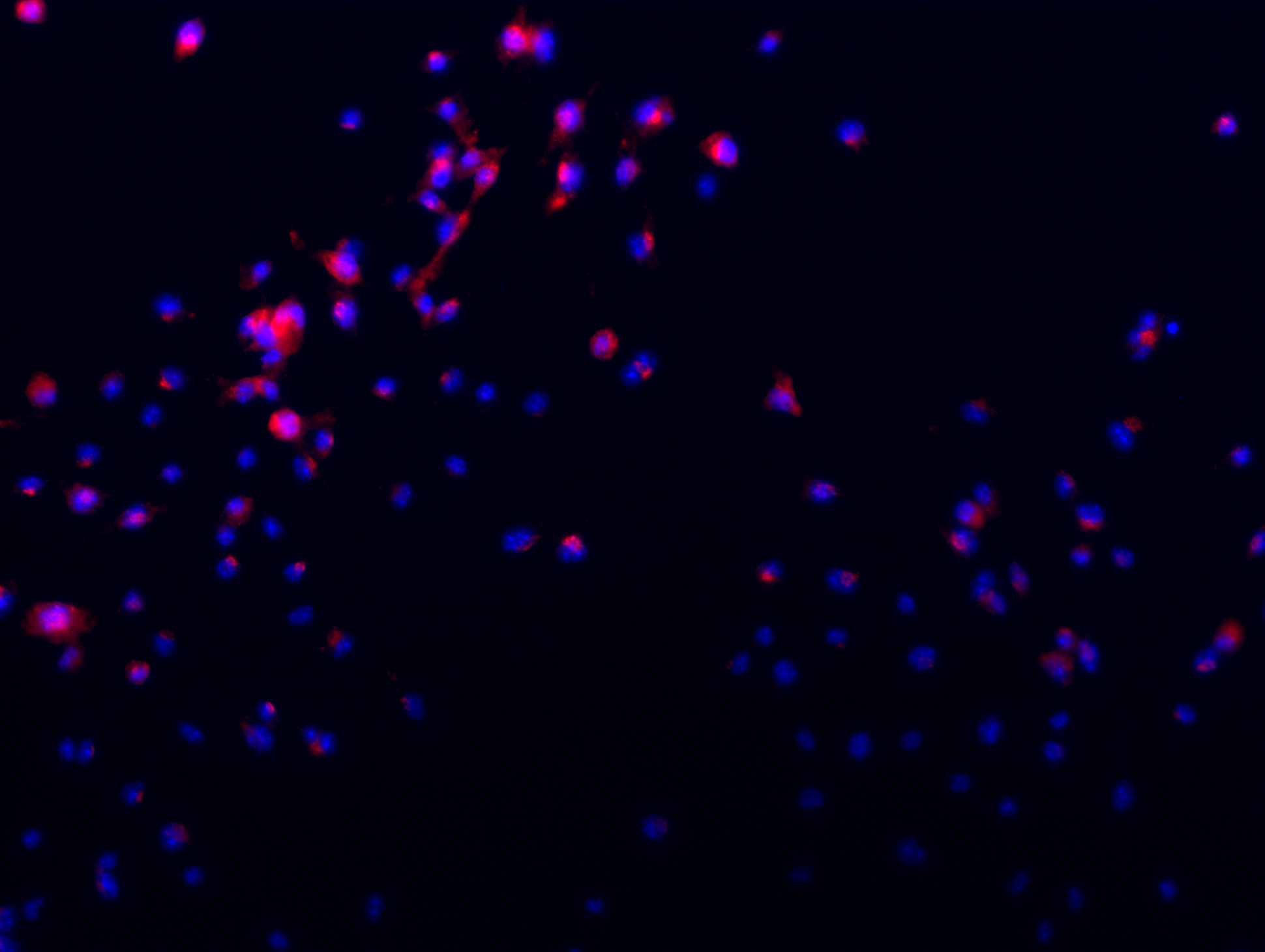

Supplement: Supplementary file 4 — Source data Fig. 4 [file 44319_2025_516_MOESM4_ESM.zip › Figure 4/4E/IF CD163 Sepp1KO IFNg.tif (RGB).tif]

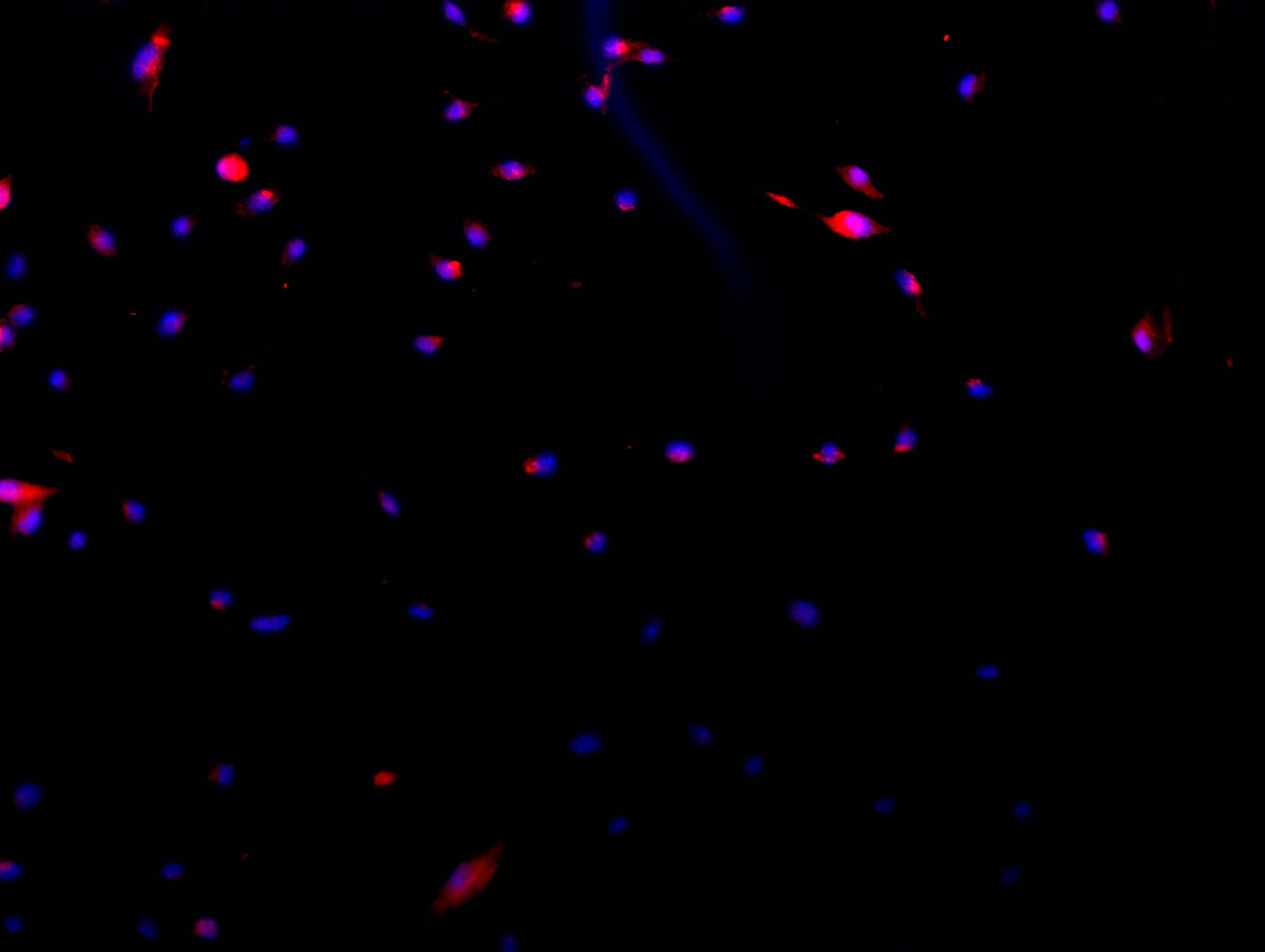

Supplement: Supplementary file 4 — Source data Fig. 4 [file 44319_2025_516_MOESM4_ESM.zip › Figure 4/4E/IF CD163 Sepp1KO IL10.tif (RGB).tif]

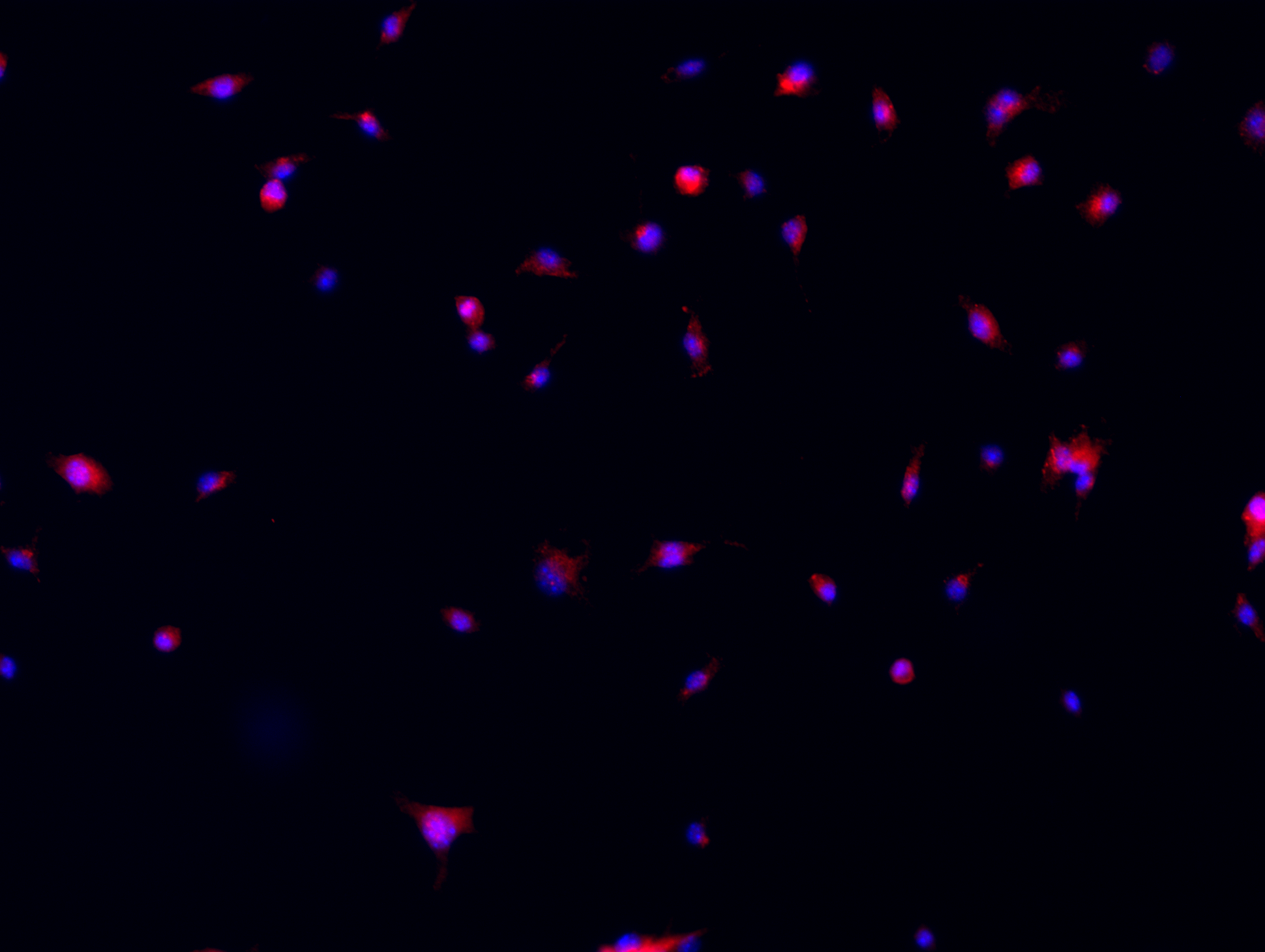

Supplement: Supplementary file 4 — Source data Fig. 4 [file 44319_2025_516_MOESM4_ESM.zip › Figure 4/4E/IF CD163 WT IFNg.tif (RGB).tif]

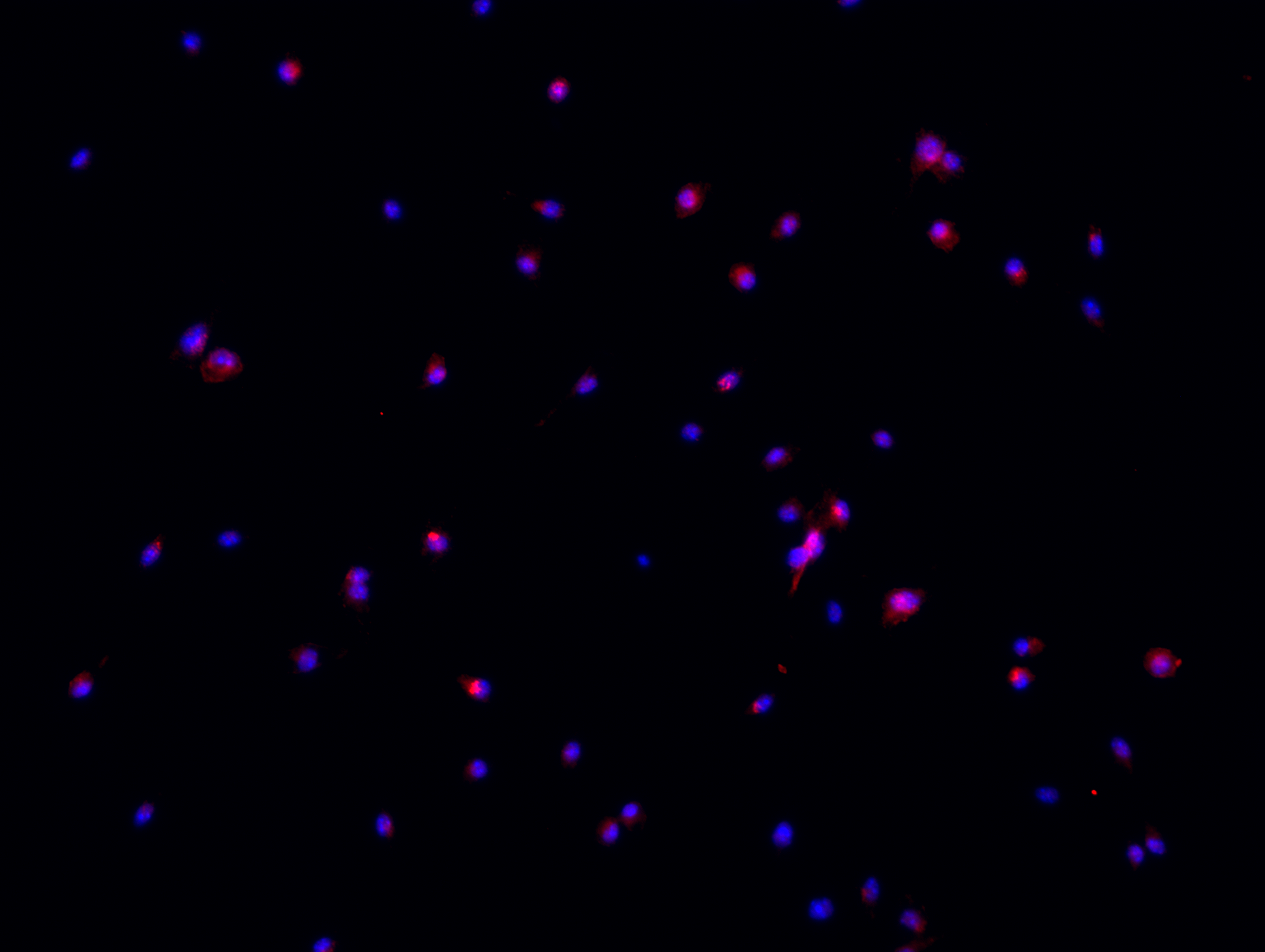

Supplement: Supplementary file 4 — Source data Fig. 4 [file 44319_2025_516_MOESM4_ESM.zip › Figure 4/4E/IF CD163 WT IL10.tif (RGB).tif]

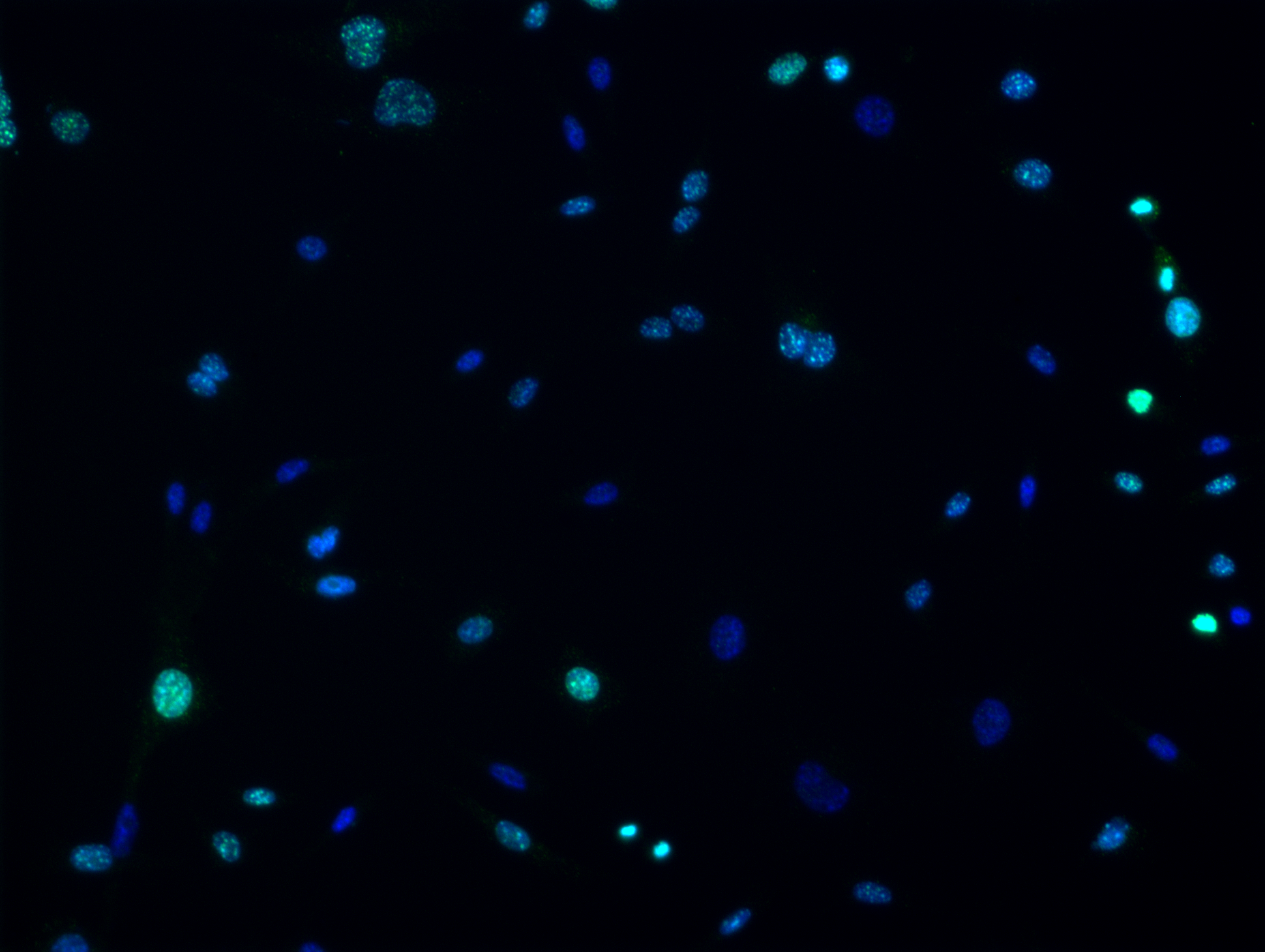

Supplement: Supplementary file 4 — Source data Fig. 4 [file 44319_2025_516_MOESM4_ESM.zip › Figure 4/4G/IF Ki67 Sepp1KO IFNg.tif (RGB).tif]

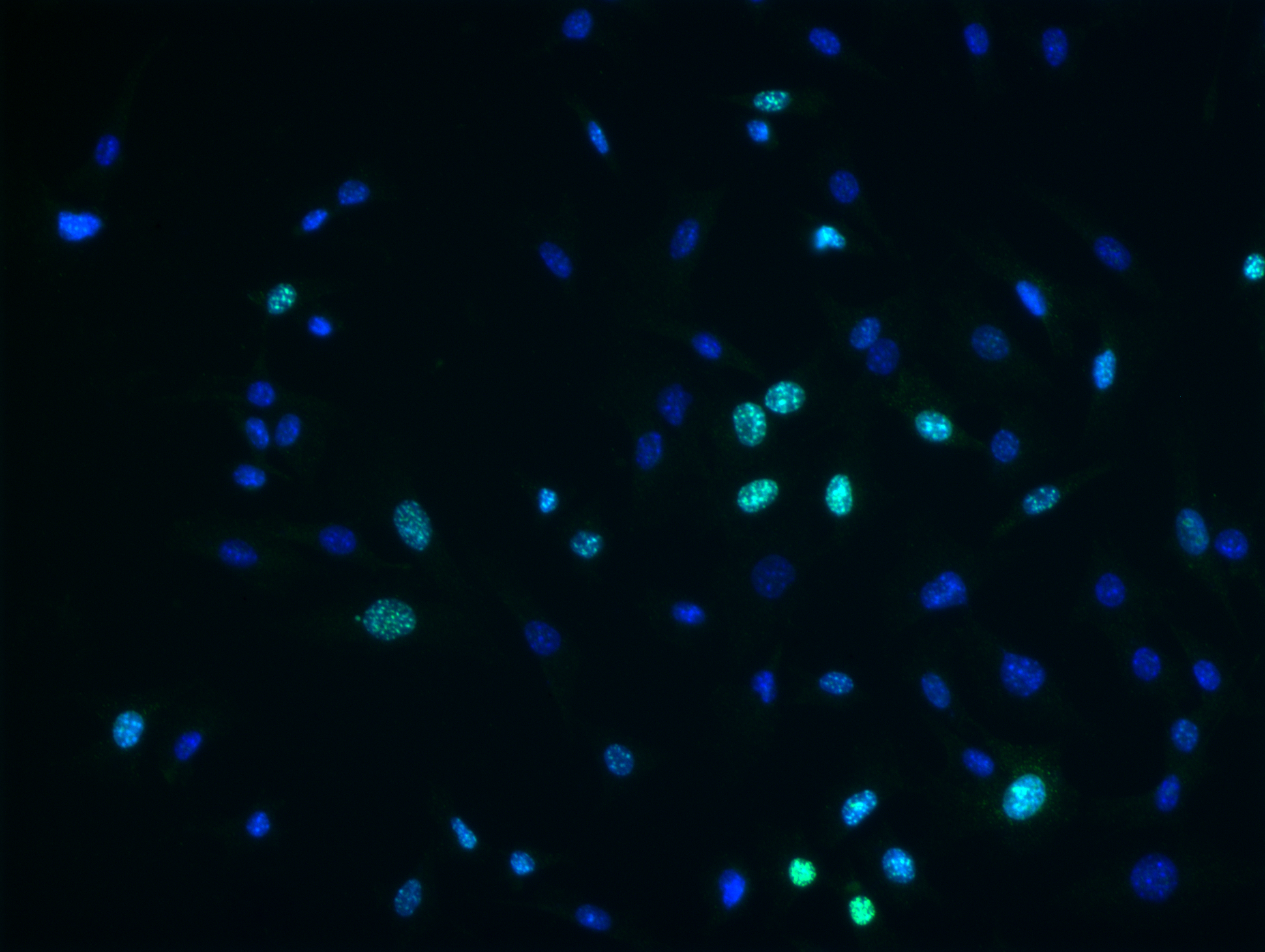

Supplement: Supplementary file 4 — Source data Fig. 4 [file 44319_2025_516_MOESM4_ESM.zip › Figure 4/4G/IF Ki67 Sepp1KO IL10.tif (RGB).tif]

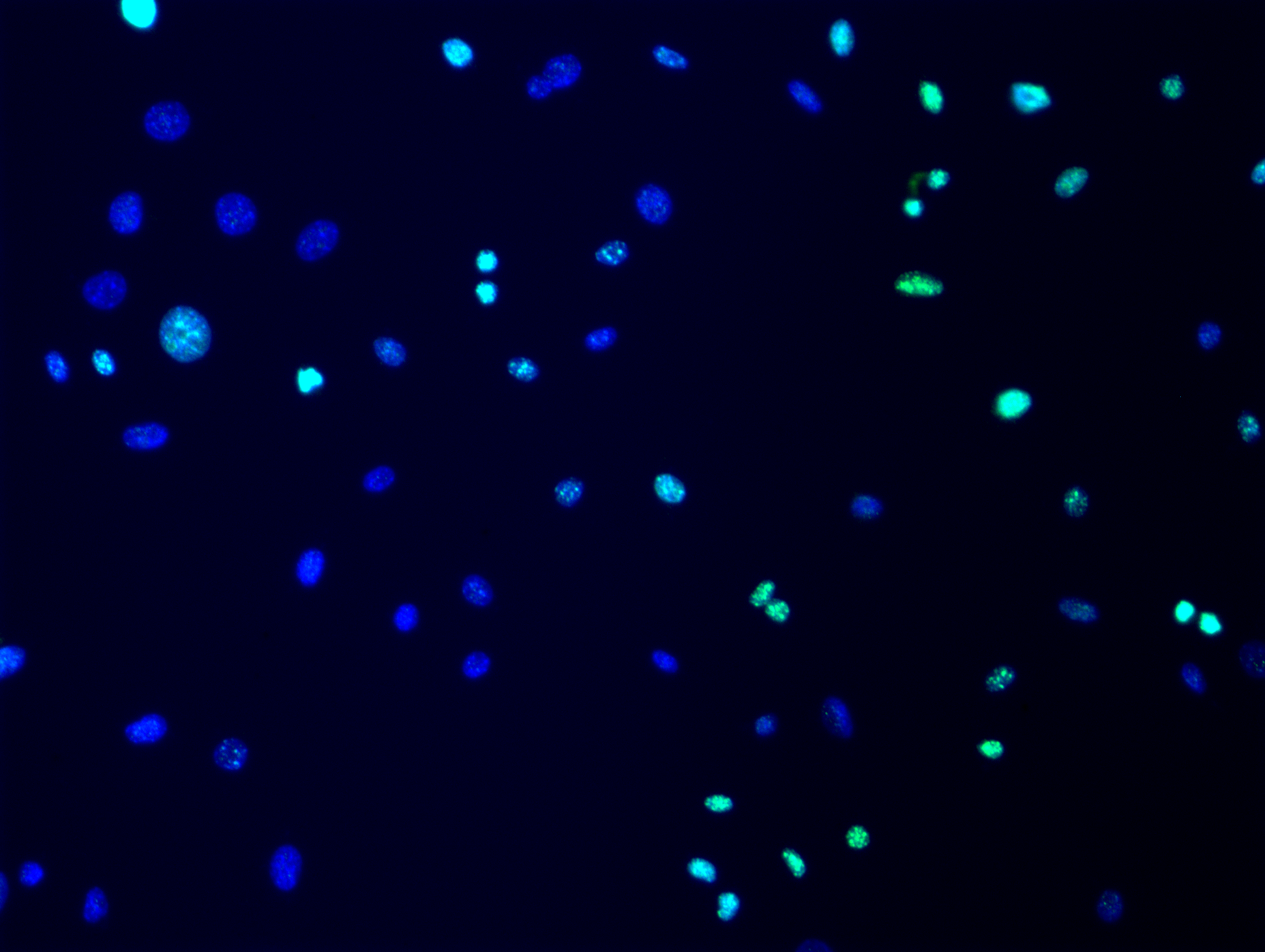

Supplement: Supplementary file 4 — Source data Fig. 4 [file 44319_2025_516_MOESM4_ESM.zip › Figure 4/4G/IF Ki67 WT IFNg.tif (RGB).tif]

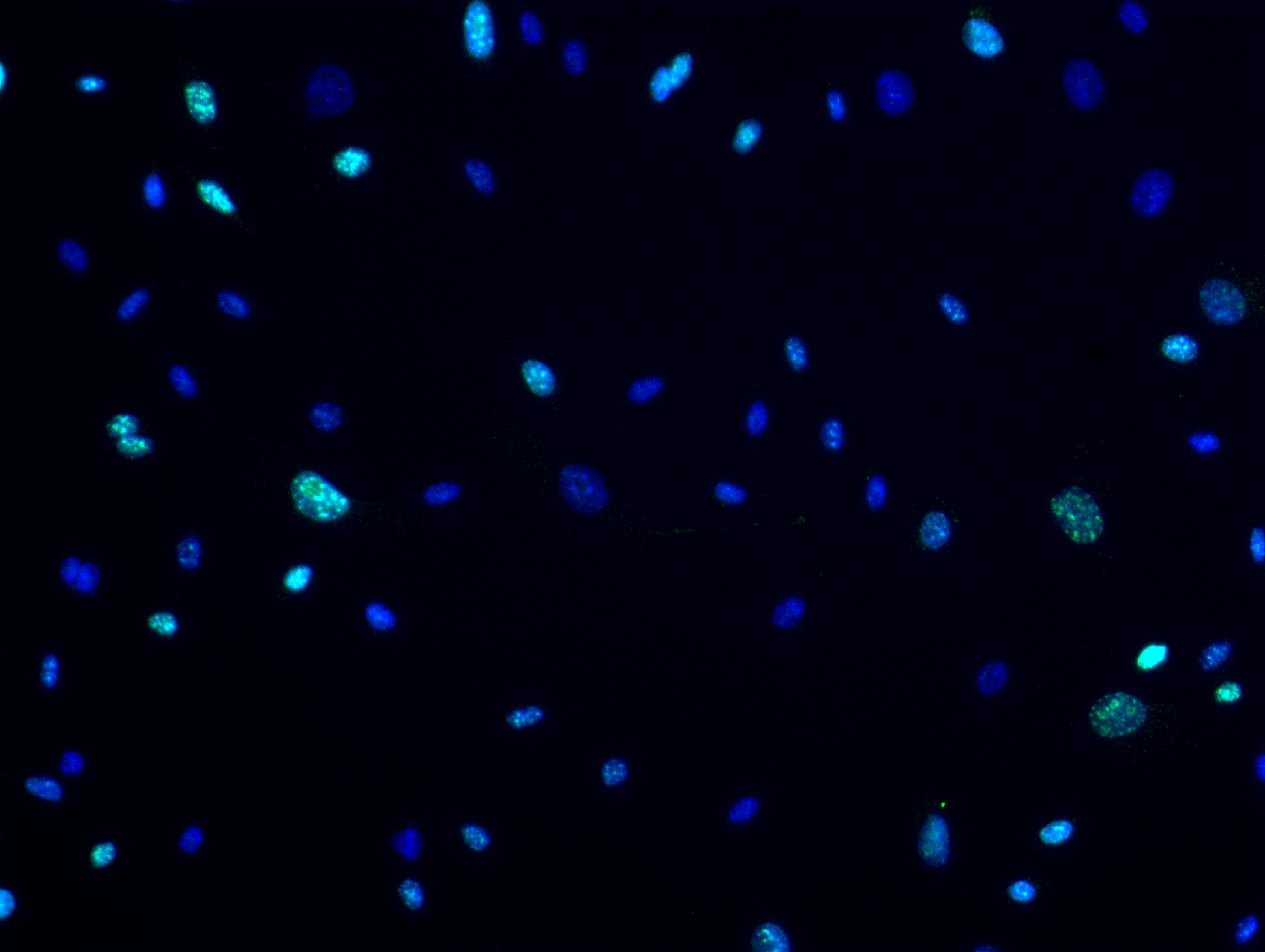

Supplement: Supplementary file 4 — Source data Fig. 4 [file 44319_2025_516_MOESM4_ESM.zip › Figure 4/4G/IF Ki67 WT IL10.tif (RGB).tif]

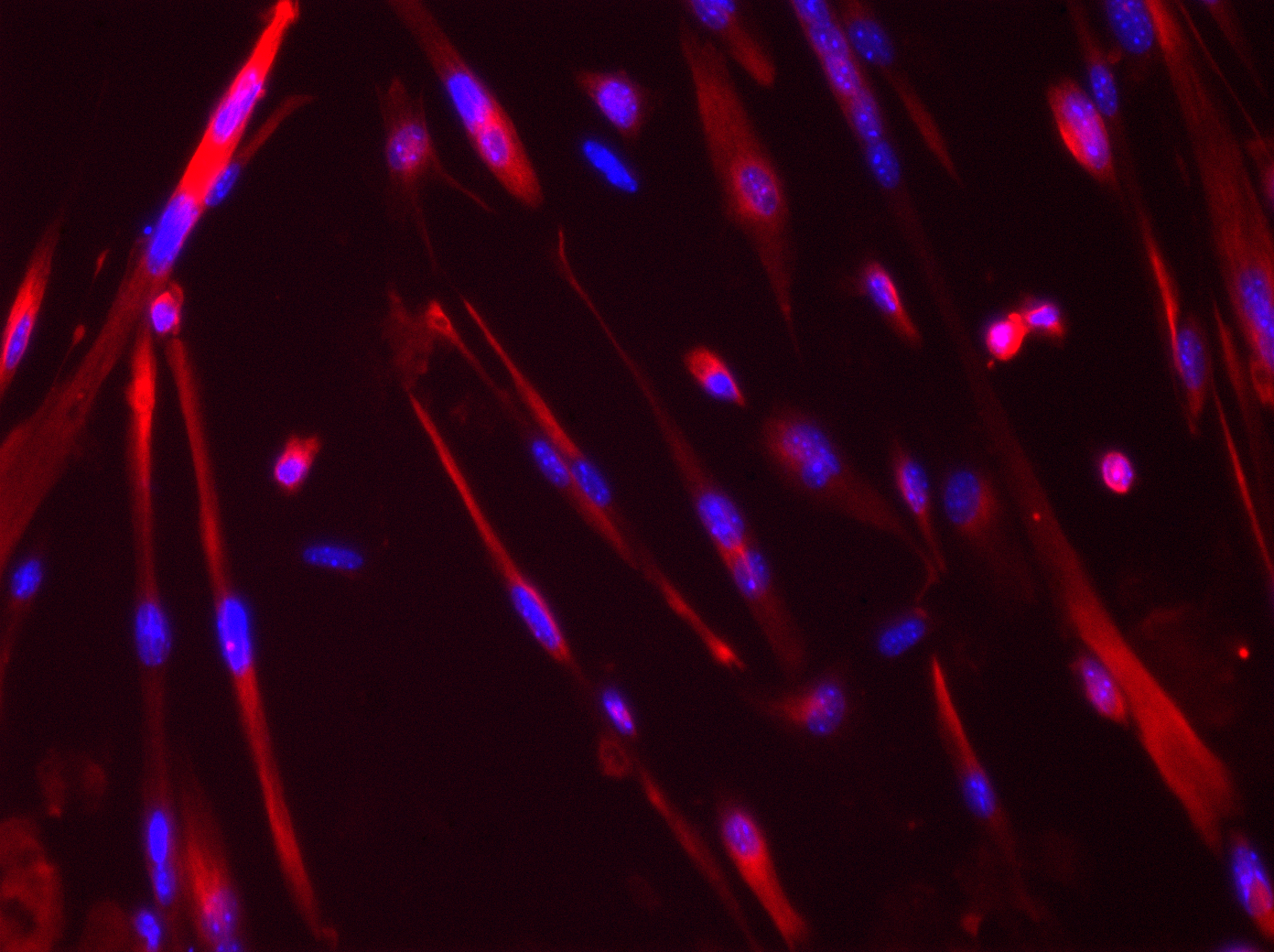

Supplement: Supplementary file 4 — Source data Fig. 4 [file 44319_2025_516_MOESM4_ESM.zip › Figure 4/4H/IF Desmin Sepp1KO IFNg.tif (RGB).tif]

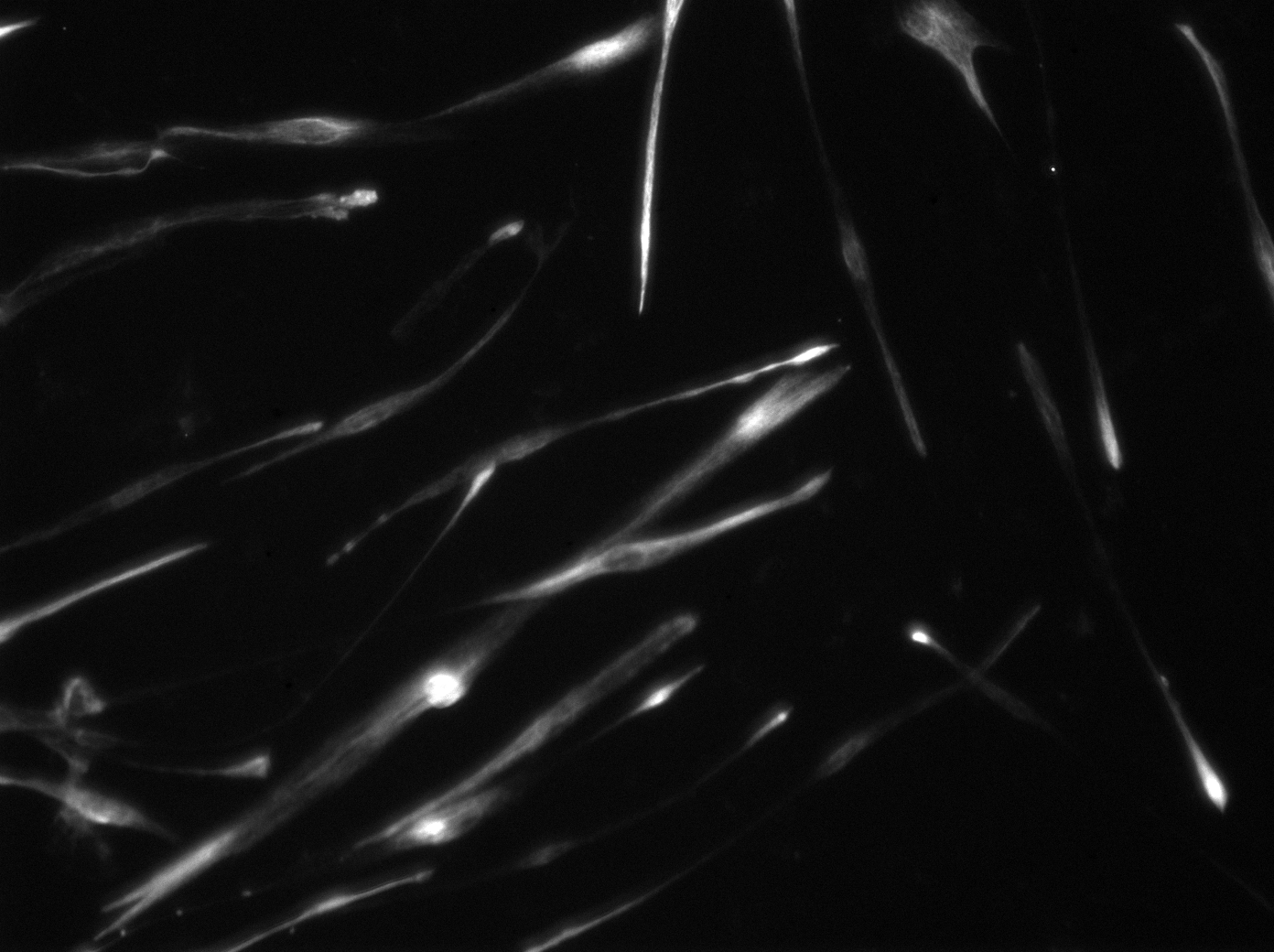

Supplement: Supplementary file 4 — Source data Fig. 4 [file 44319_2025_516_MOESM4_ESM.zip › Figure 4/4H/IF Desmin WT IFNg.tif]

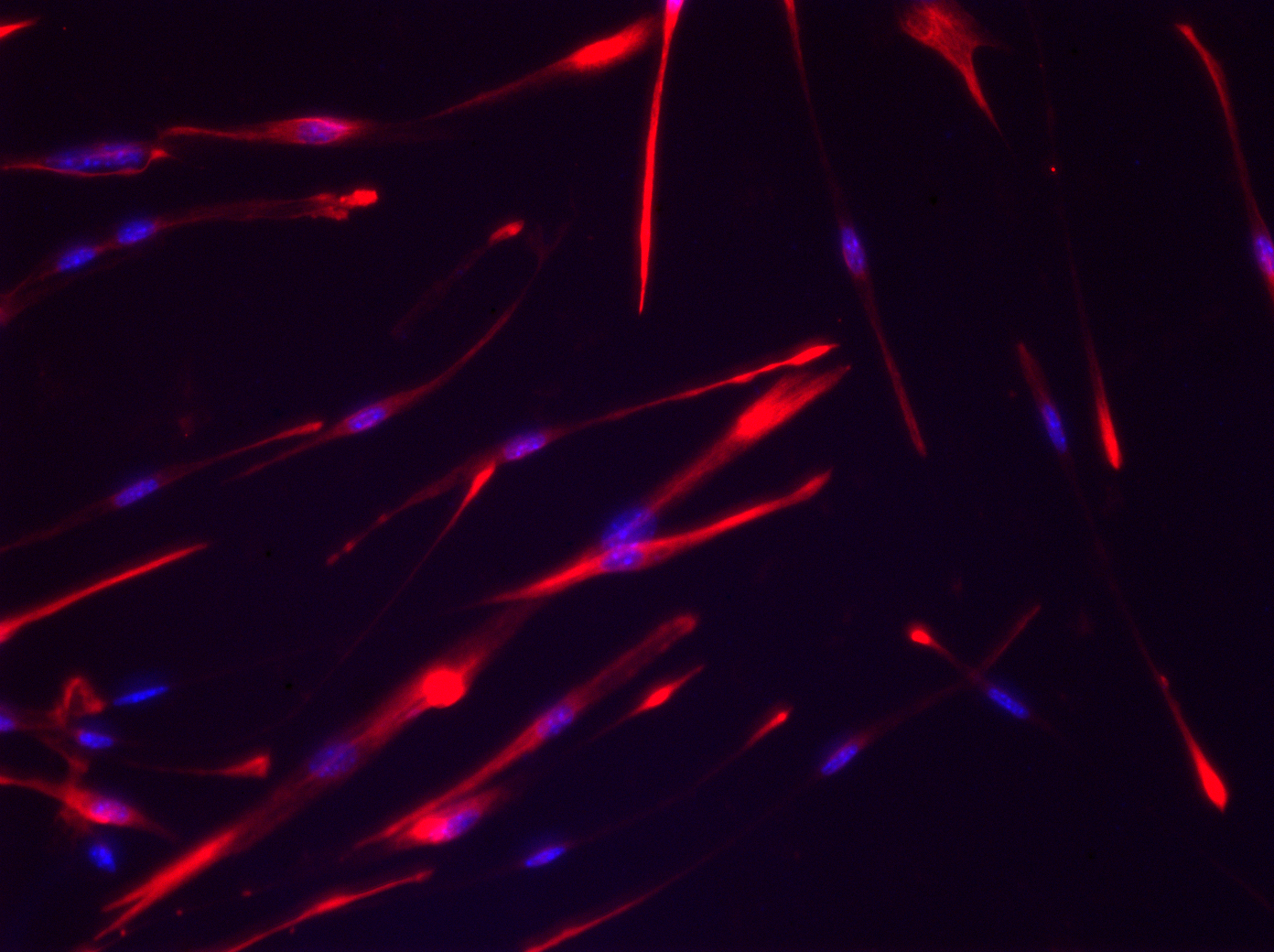

Supplement: Supplementary file 4 — Source data Fig. 4 [file 44319_2025_516_MOESM4_ESM.zip › Figure 4/4H/IF Desmin WT IFNg.tif (RGB).tif]

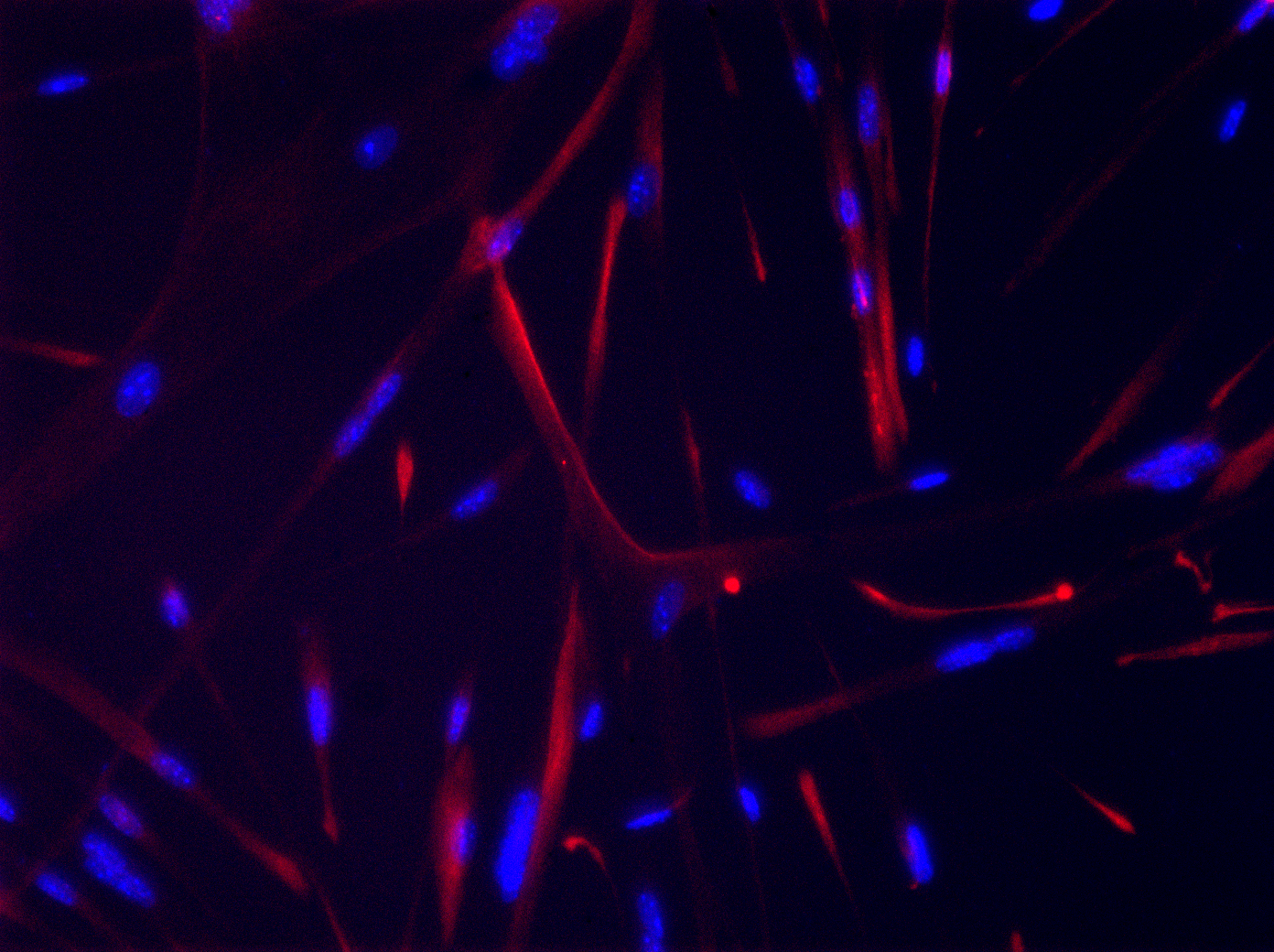

Supplement: Supplementary file 4 — Source data Fig. 4 [file 44319_2025_516_MOESM4_ESM.zip › Figure 4/4H/IF Desmin WT IL10.tif (RGB).tif]

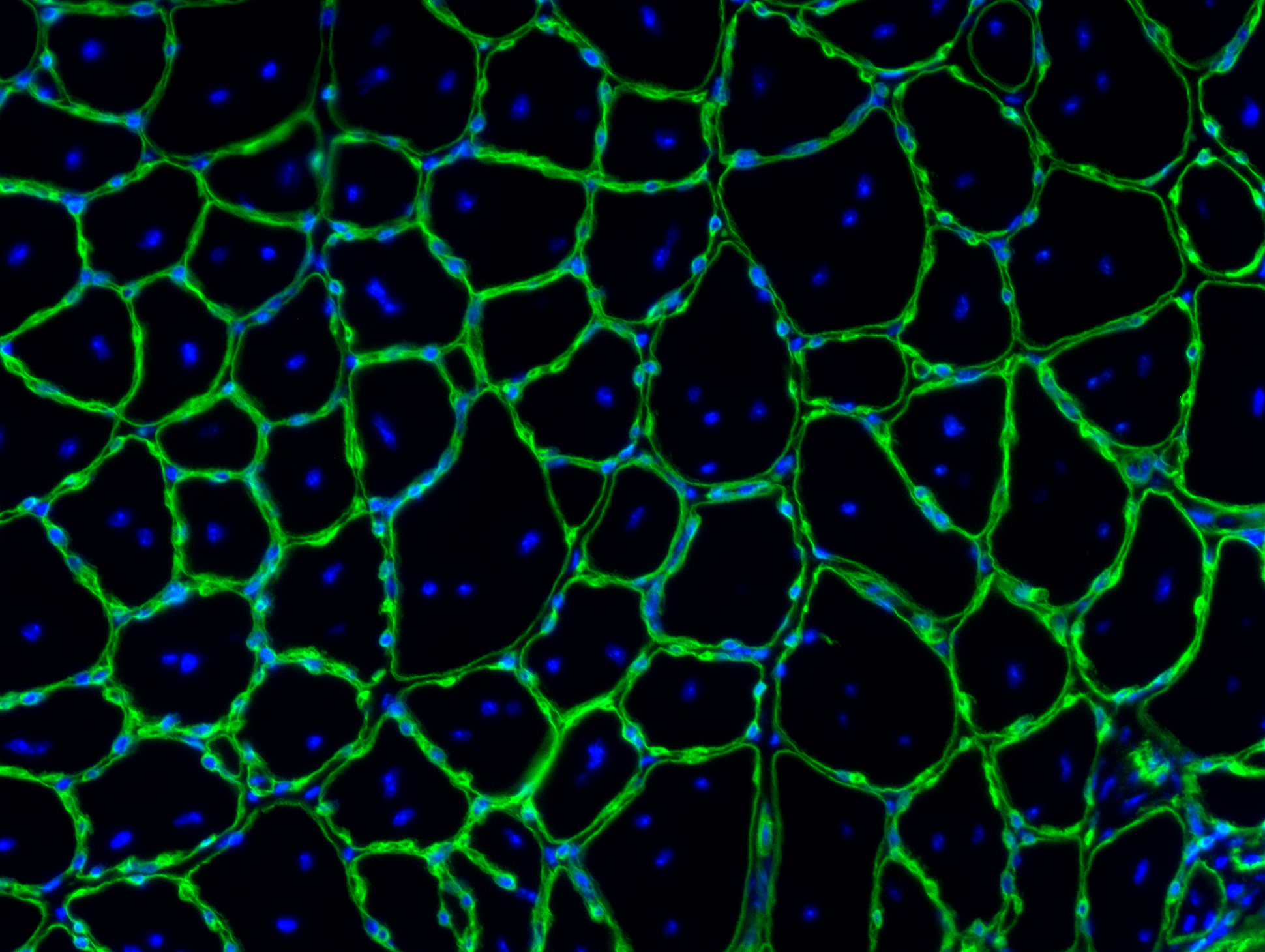

Supplement: Supplementary file 5 — Source data Fig. 5 [file 44319_2025_516_MOESM5_ESM.zip › Figure 5/5D/IF Laminin Sepp1+.tif (RGB).tif]

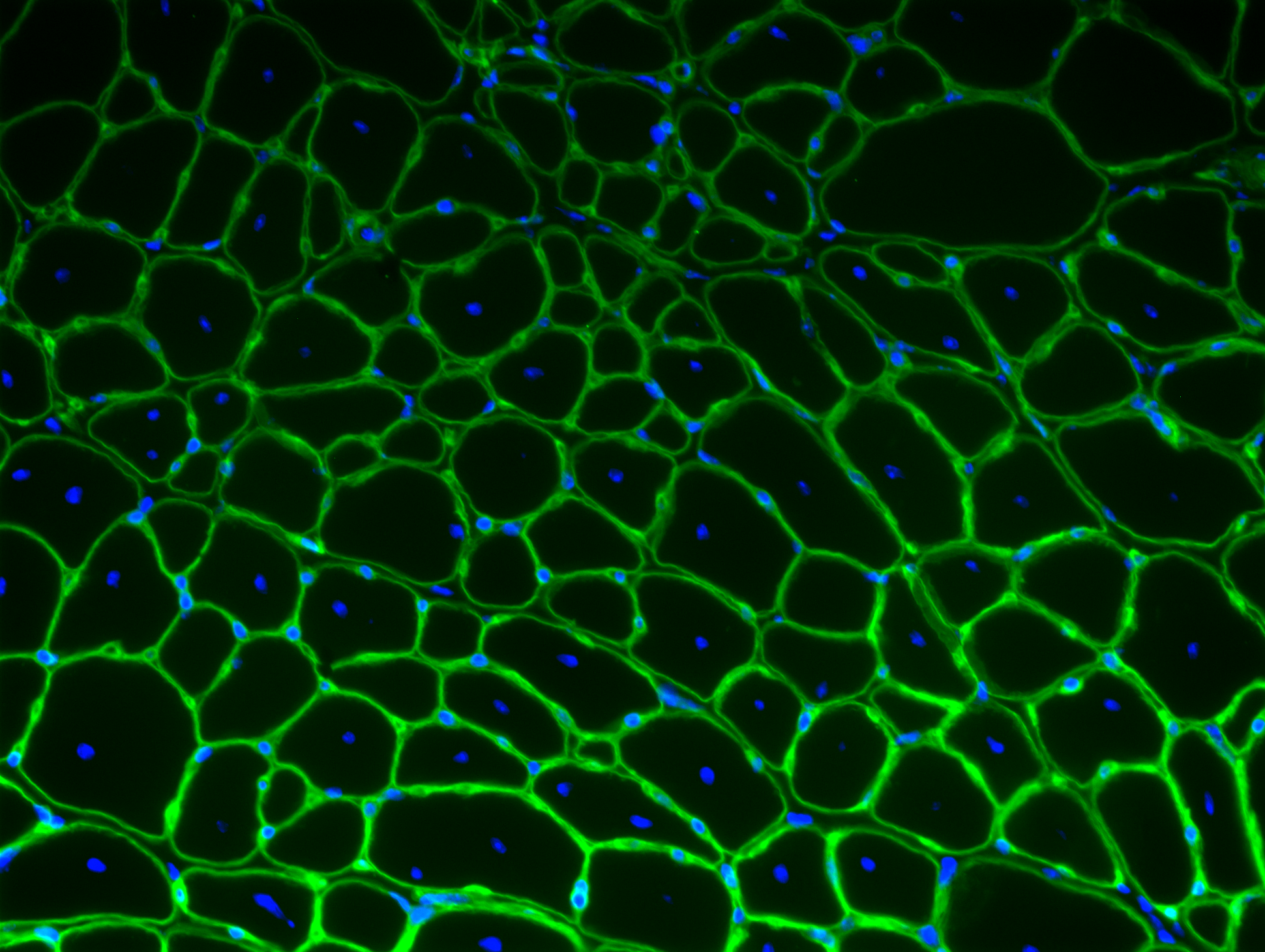

Supplement: Supplementary file 5 — Source data Fig. 5 [file 44319_2025_516_MOESM5_ESM.zip › Figure 5/5D/IF Laminin Sepp1DMac.tif (RGB).tif]

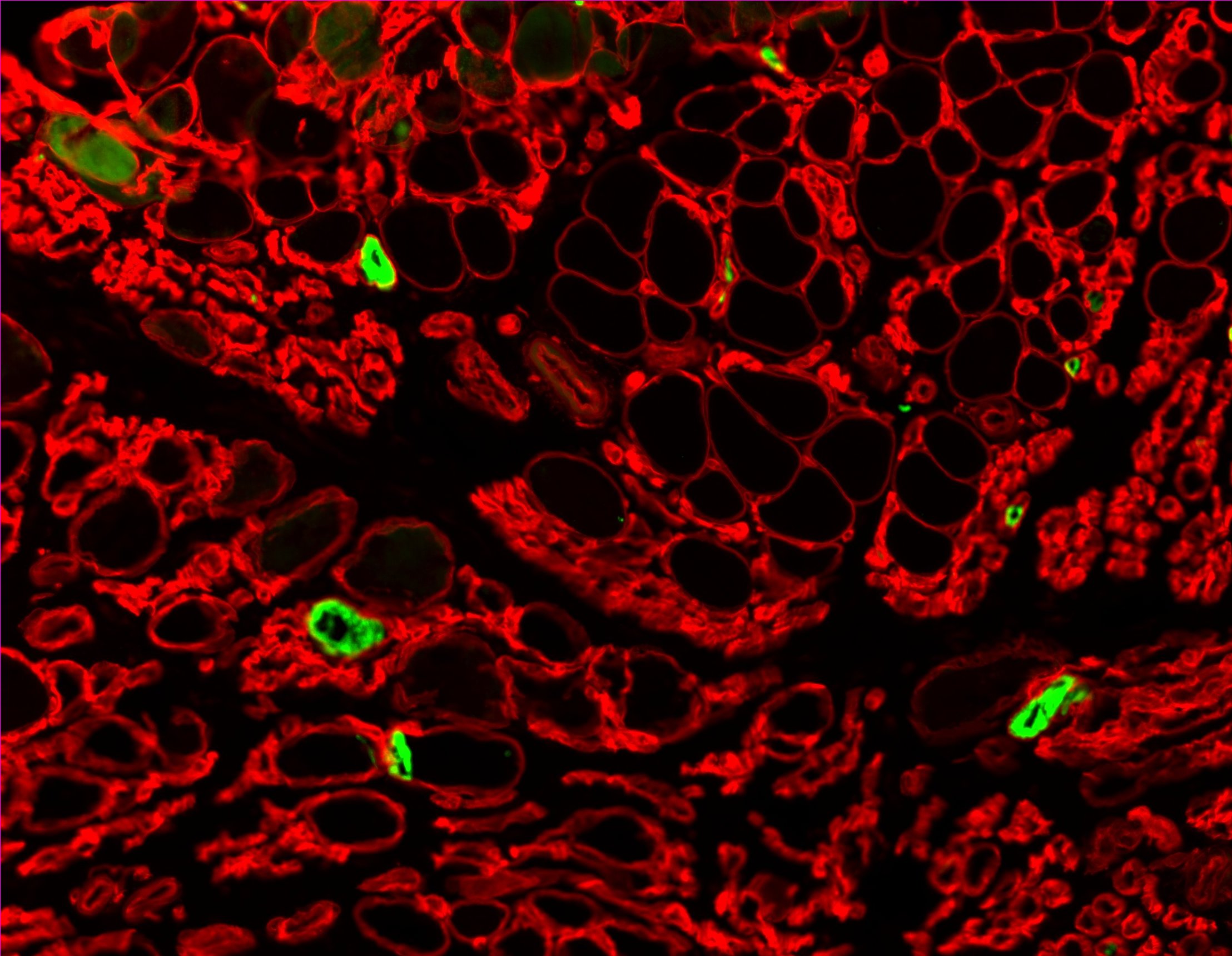

Supplement: Supplementary file 5 — Source data Fig. 5 [file 44319_2025_516_MOESM5_ESM.zip › Figure 5/5F/Old eMHC RGB.jpg]

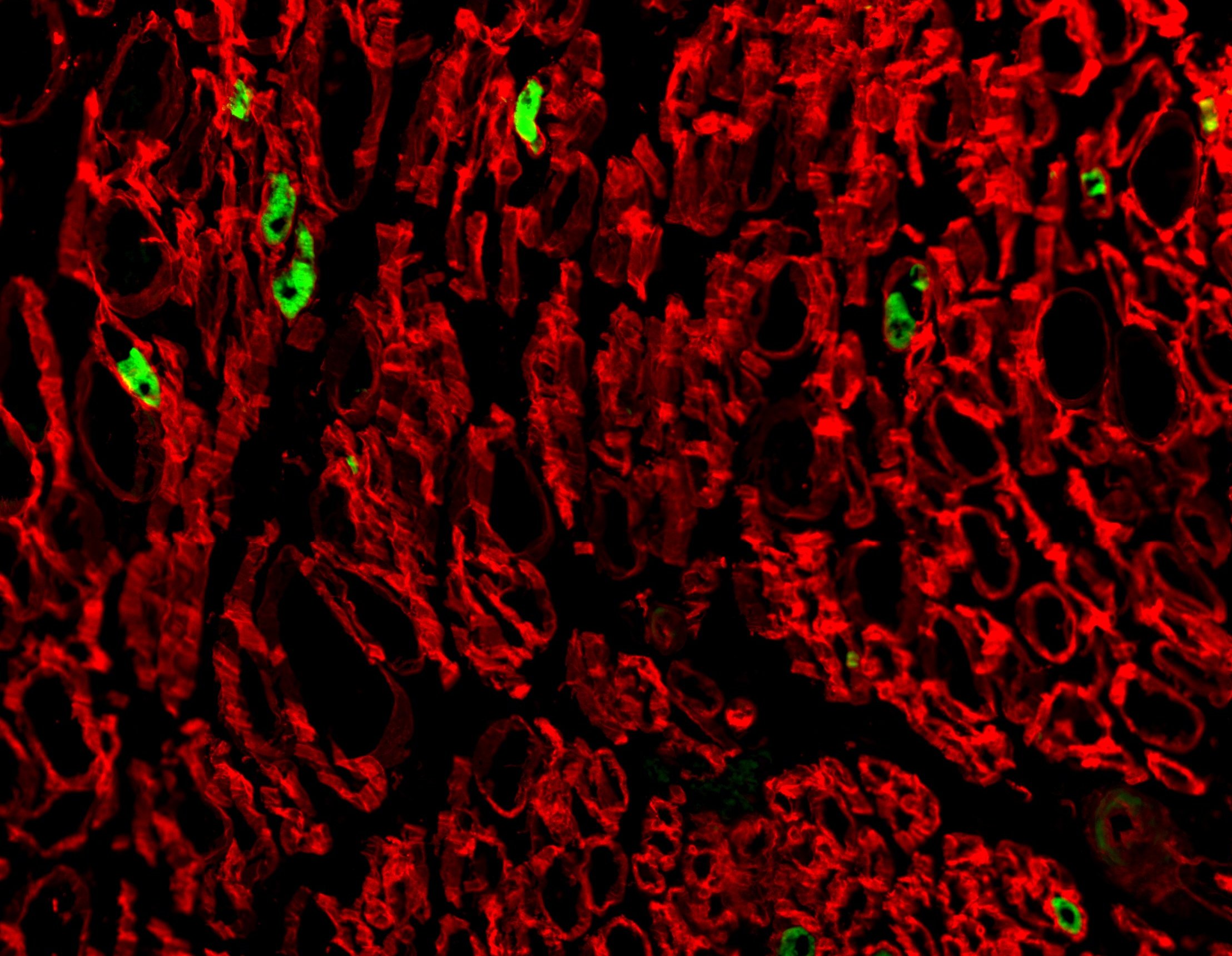

Supplement: Supplementary file 5 — Source data Fig. 5 [file 44319_2025_516_MOESM5_ESM.zip › Figure 5/5F/SeppKO eMHC RGB.jpg]

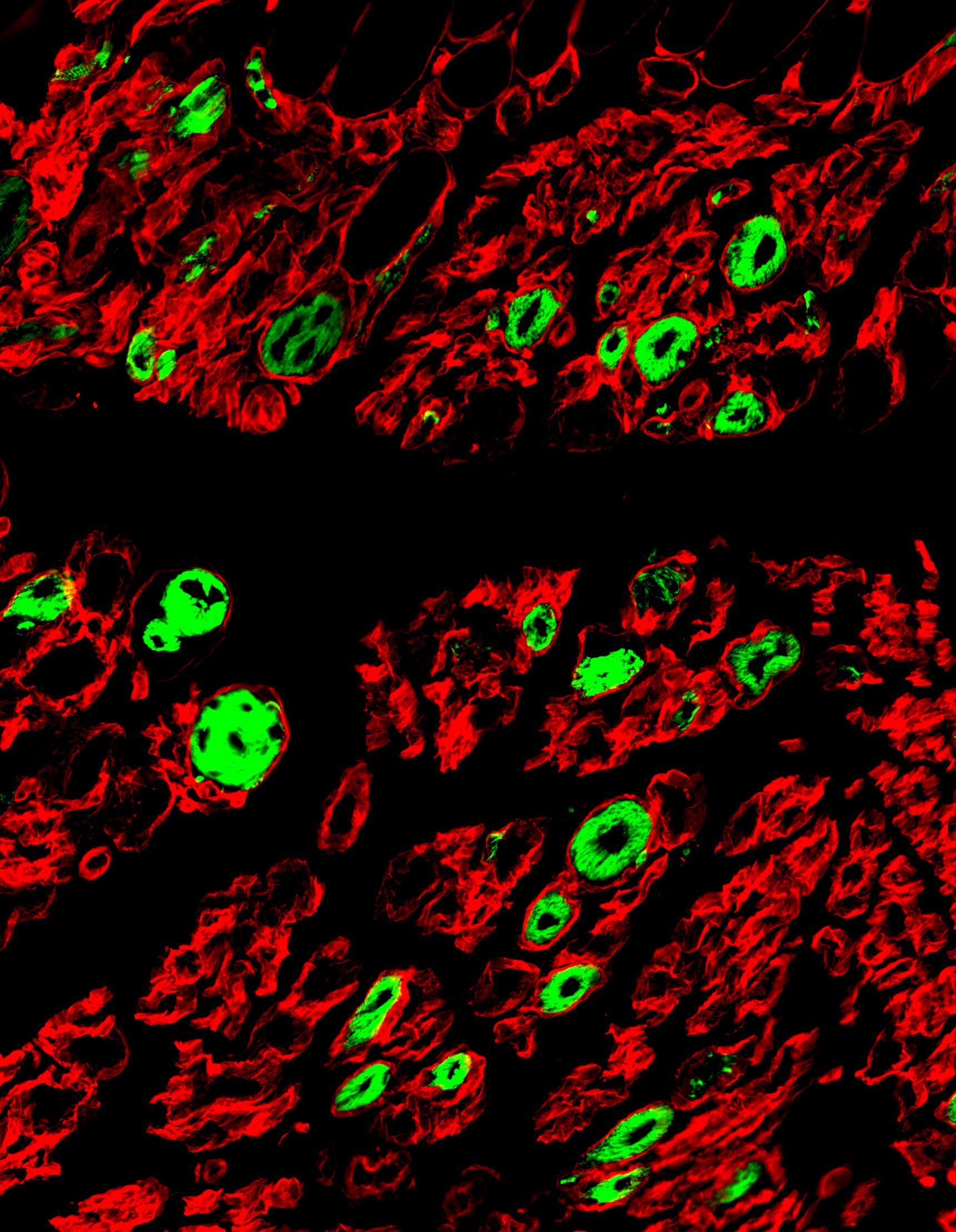

Supplement: Supplementary file 5 — Source data Fig. 5 [file 44319_2025_516_MOESM5_ESM.zip › Figure 5/5F/Young eMHC RGB.jpg]

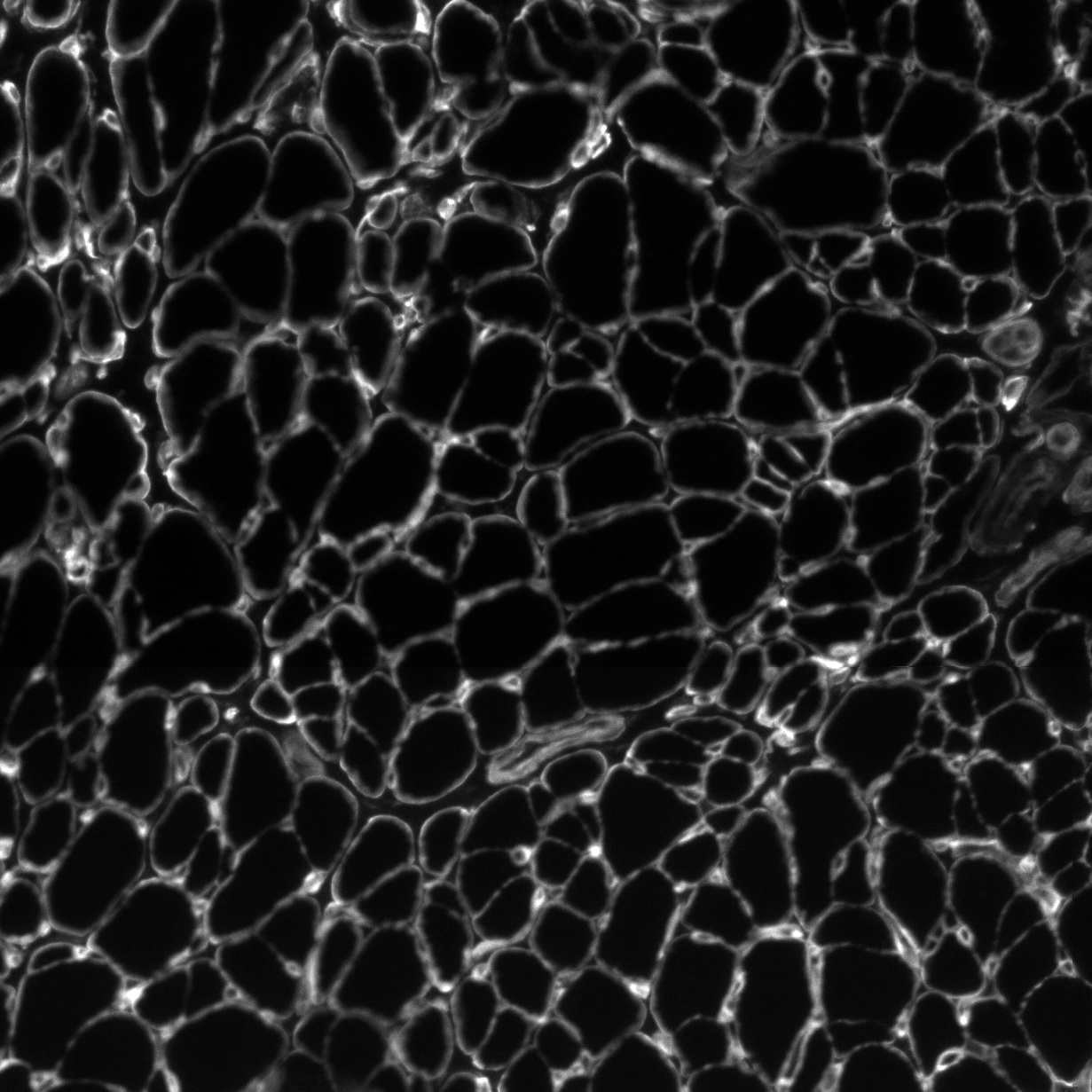

Supplement: Supplementary file 5 — Source data Fig. 5 [file 44319_2025_516_MOESM5_ESM.zip › Figure 5/5G/IF Laminin Old.tif]

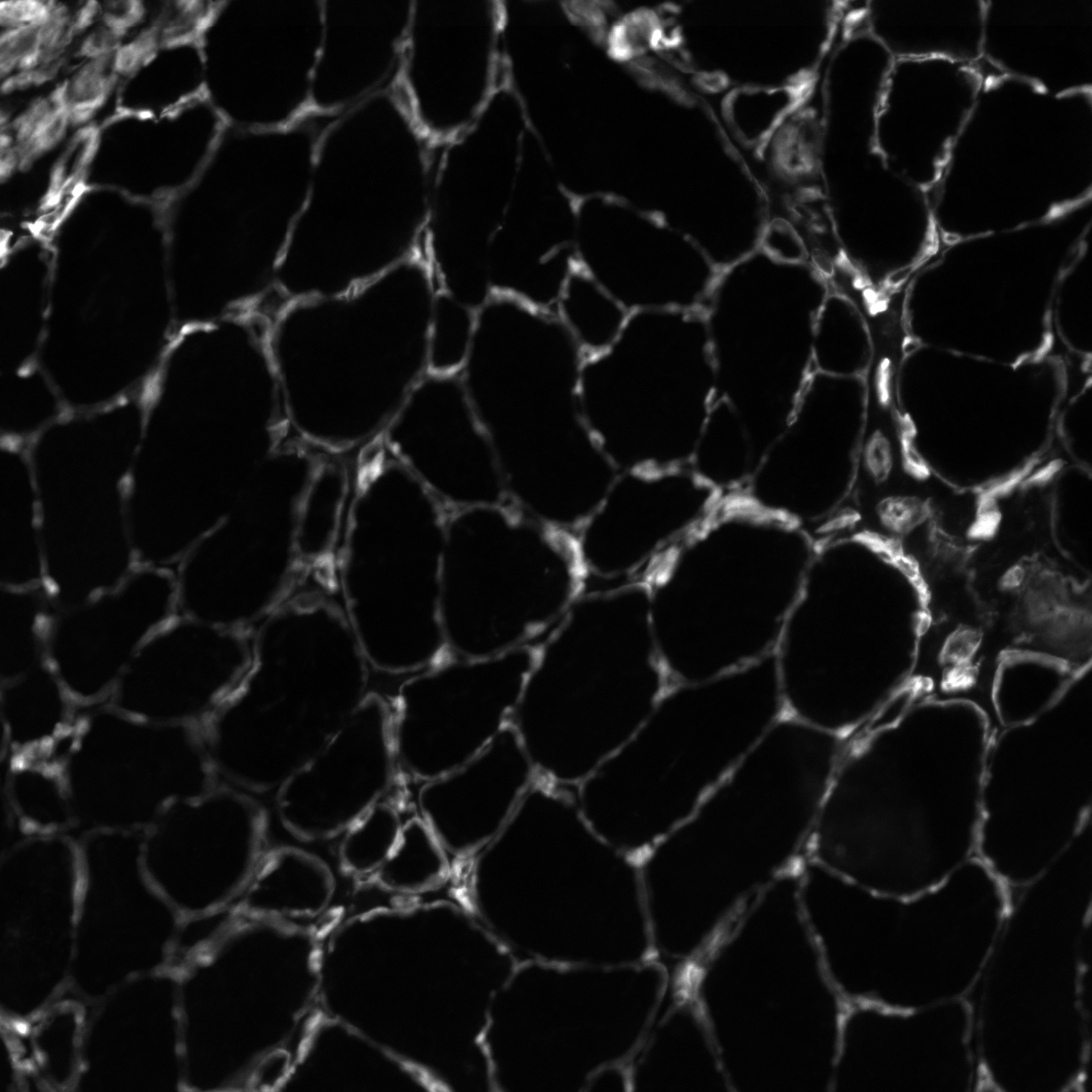

Supplement: Supplementary file 5 — Source data Fig. 5 [file 44319_2025_516_MOESM5_ESM.zip › Figure 5/5G/IF Laminin Sepp1DMac.tif]

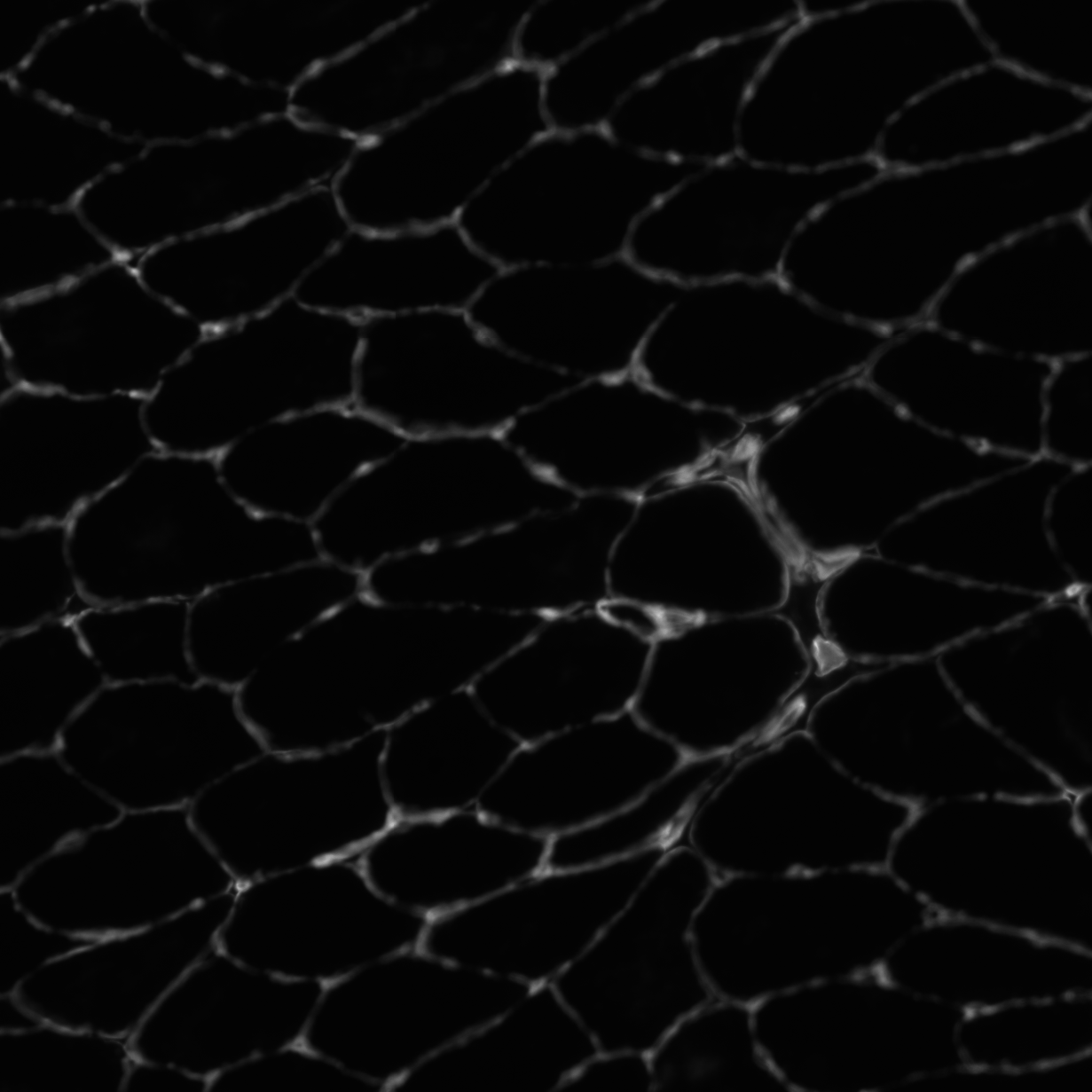

Supplement: Supplementary file 5 — Source data Fig. 5 [file 44319_2025_516_MOESM5_ESM.zip › Figure 5/5G/IF Laminin Young.tif]
