# Supplementary material for: Immune aging impairs muscle regeneration via macrophage-derived anti-oxidant selenoprotein P
Source: EMBO Rep. 2025 Jul 18;26(16):4153–79. doi: 10.1038/s44319-025-00516-3 (PMC12373998; doi:10.1038/s44319-025-00516-3)
Supplement: Supplementary file 6 — Expanded View Figures [file 44319_2025_516_MOESM6_ESM.pdf]

Expanded View Figures

Figure EV1. Histological analysis of regenerating young and old muscle.

Tibialis Anterior muscles from young (10 weeks old) and old (24 months old) mice were injected or not with cardiotoxin and were harvested 2, 4, 7 and 28 days after injury. (**A**) Mouse body weight was quantified ($n = 3$–9). Two-way ANOVA test was non significant. Multiple unpaired t tests were performed and the P values are given for each day. (**B–I**) The muscle sections were immunostained for various proteins. From laminin immunostaining, the cross-section myofiber area distribution at day 28 after injury (**B**) ($n = 6$), and the total muscle area (**C**) ($n = 4$–6) were measured. Two-way ANOVA test was performed followed by multiple comparisons using Šidák test. (**B**) Multiple unpaired t tests were additionally performed and the P values are shown in blue. Representative pictures of immunostainings for Laminin (**C**) (bars = 80 μm), IgGs (**E**) (bars = 50 μm), PDGFRα (**F**) (bars = 50 μm), Collagen I (**G**) (bars = 40 μm), CD31 (**H**) (bars = 40 μm), and F4/80 (**I**) (bars = 40 μm). Data information: Values are given as mean ± SEM. Each dot represents one mouse. Result of the two-way ANOVA test is shown for each graph as *$P < 0.05$; ****$P < 0.0001$.

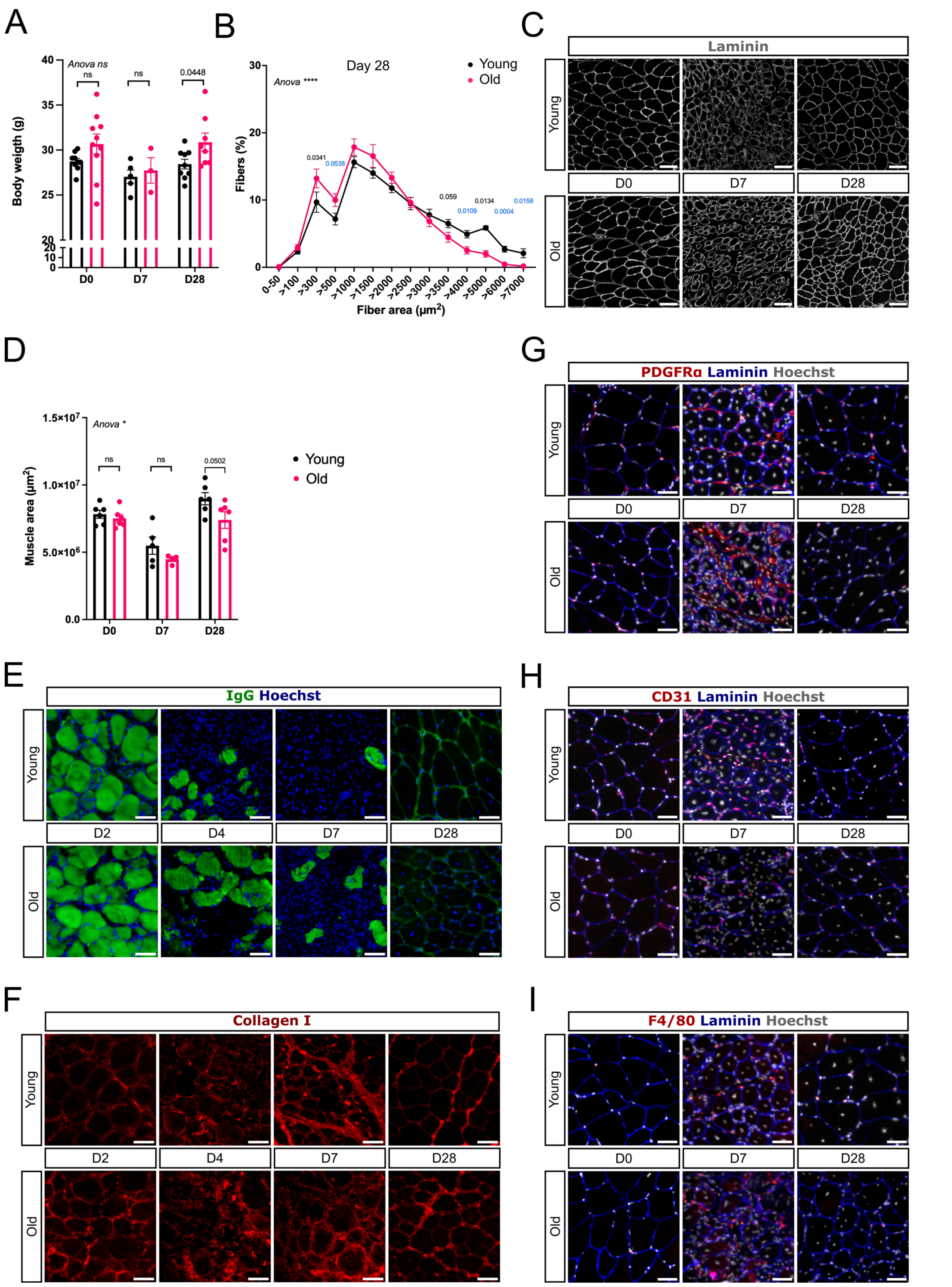
A
Anova ns
ns
ns
0.0448
Body weigth (g)
D0
D7
D28
B
Day 28
Anova ****
Young
Old
Fibers (%)
Fiber area (μm2)
C
Laminin
Young
Old
D0
D7
D28
D
Anova *
ns
ns
0.0502
Muscle area (μm2)
Young
Old
E
IgG Hoechst
D2
D4
D7
D28
F
Collagen I
G
PDGFRα Laminin Hoechst
H
CD31 Laminin Hoechst
I
F4/80 Laminin Hoechst

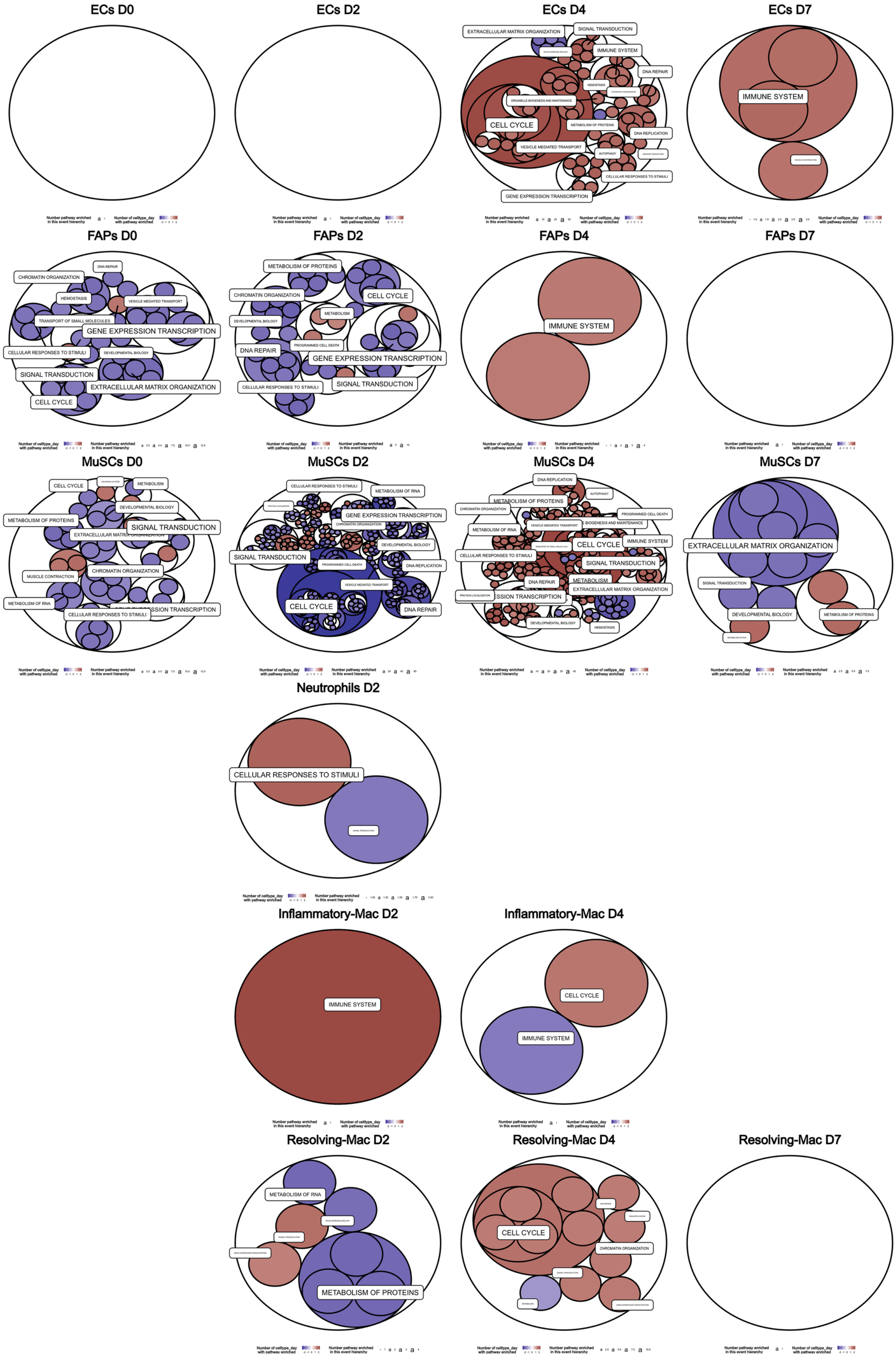
ECs D0
ECs D2
ECs D4
EXTRACELLULAR MATRIX ORGANIZATION
SIGNAL TRANSDUCTION
IMMUNE SYSTEM
DNA REPAIR
HEMOSTASIS
CELL CYCLE
METABOLISM OF PROTEINS
DNA REPLICATION
VESICLE MEDIATED TRANSPORT
AUTOPHAGY
CELLULAR RESPONSES TO STIMULI
GENE EXPRESSION TRANSCRIPTION
ECs D7
IMMUNE SYSTEM
FAPs D0
DNA REPAIR
CHROMATIN ORGANIZATION
HEMOSTASIS
VESICLE MEDIATED TRANSPORT
TRANSPORT OF SMALL MOLECULES
GENE EXPRESSION TRANSCRIPTION
CELLULAR RESPONSES TO STIMULI
DEVELOPMENTAL BIOLOGY
SIGNAL TRANSDUCTION
EXTRACELLULAR MATRIX ORGANIZATION
CELL CYCLE
FAPs D2
METABOLISM OF PROTEINS
CHROMATIN ORGANIZATION
CELL CYCLE
METABOLISM
DEVELOPMENTAL BIOLOGY
PROGRAMMED CELL DEATH
DNA REPAIR
GENE EXPRESSION TRANSCRIPTION
CELLULAR RESPONSES TO STIMULI
SIGNAL TRANSDUCTION
FAPs D4
IMMUNE SYSTEM
FAPs D7
MuSCs D0
CELL CYCLE
METABOLISM
DEVELOPMENTAL BIOLOGY
METABOLISM OF PROTEINS
SIGNAL TRANSDUCTION
MUSCLE CONTRACTION
CHROMATIN ORGANIZATION
METABOLISM OF RNA
CELLULAR RESPONSES TO STIMULI
MuSCs D2
CELLULAR RESPONSES TO STIMULI
METABOLISM OF RNA
GENE EXPRESSION TRANSCRIPTION
CHROMATIN ORGANIZATION
DEVELOPMENTAL BIOLOGY
SIGNAL TRANSDUCTION
PROGRAMMED CELL DEATH
DNA REPLICATION
VESICLE MEDIATED TRANSPORT
CELL CYCLE
DNA REPAIR
MuSCs D4
DNA REPLICATION
AUTOPHAGY
METABOLISM OF PROTEINS
CHROMATIN ORGANIZATION
PROGRAMMED CELL DEATH
METABOLISM OF RNA
VESICLE MEDIATED TRANSPORT
IMMUNE SYSTEM
CELL CYCLE
CELLULAR RESPONSES TO STIMULI
SIGNAL TRANSDUCTION
DNA REPAIR
METABOLISM
EXTRACELLULAR MATRIX ORGANIZATION
PROTEIN LOCALIZATION
DEVELOPMENTAL BIOLOGY
HEMOSTASIS
MuSCs D7
EXTRACELLULAR MATRIX ORGANIZATION
SIGNAL TRANSDUCTION
DEVELOPMENTAL BIOLOGY
METABOLISM OF PROTEINS
Neutrophils D2
CELLULAR RESPONSES TO STIMULI
Inflammatory-Mac D2
IMMUNE SYSTEM
Inflammatory-Mac D4
CELL CYCLE
IMMUNE SYSTEM
Resolving-Mac D2
METABOLISM OF RNA
METABOLISM OF PROTEINS
Resolving-Mac D4
CELL CYCLE
CHROMATIN ORGANIZATION
Resolving-Mac D7
Number pathway enriched in this event hierarchy
Number of celltype_day with pathway enriched

Figure EV2. Enriched signaling pathways in old *versus* young mononucleated cells.

Hierarchical overview of Reactome pathway is presented, pathway labels correspond to 25 headers of the hierarchical levels, and the size is scaled based on the number of enriched pathways found in their respective sons. Each circle corresponds to a pathway and its color is the NES.

© The Author(s)

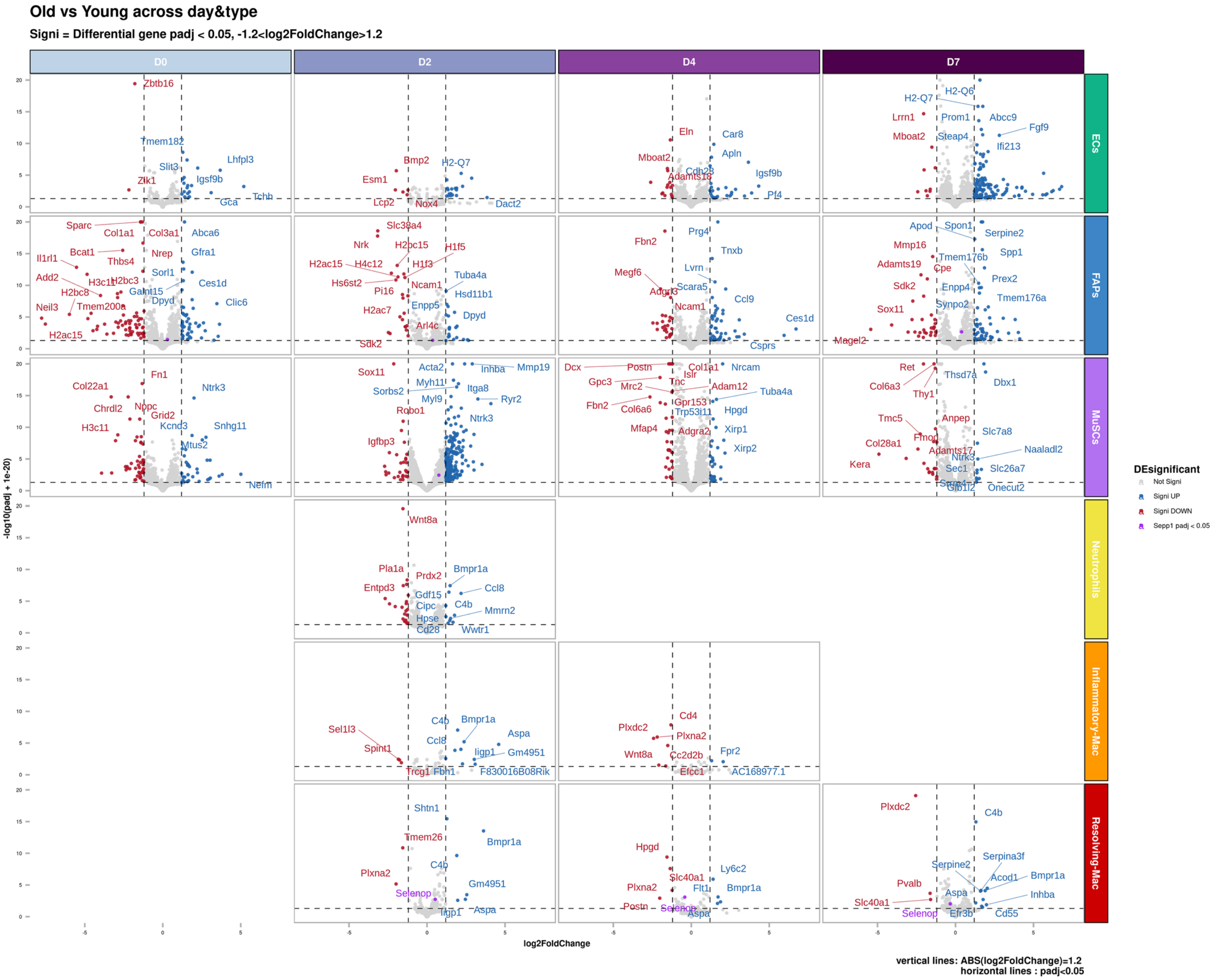

Figure EV3. Differentially expressed genes (DEG) in old *versus* young mononucleated cells.

Volcano plot showing log2 fold change (RNAseq) for old *versus* young samples plotted against the –log10 adjusted *P* value (FDR = 0.05) as determined by DESeq2. Significantly differentially expressed genes (DEG) for the Old vs Young contrast were selected by fixing a Benjamini–Hochberg corrected p-value threshold of 0.05 (padj <= 0.05) (*n* = 3).

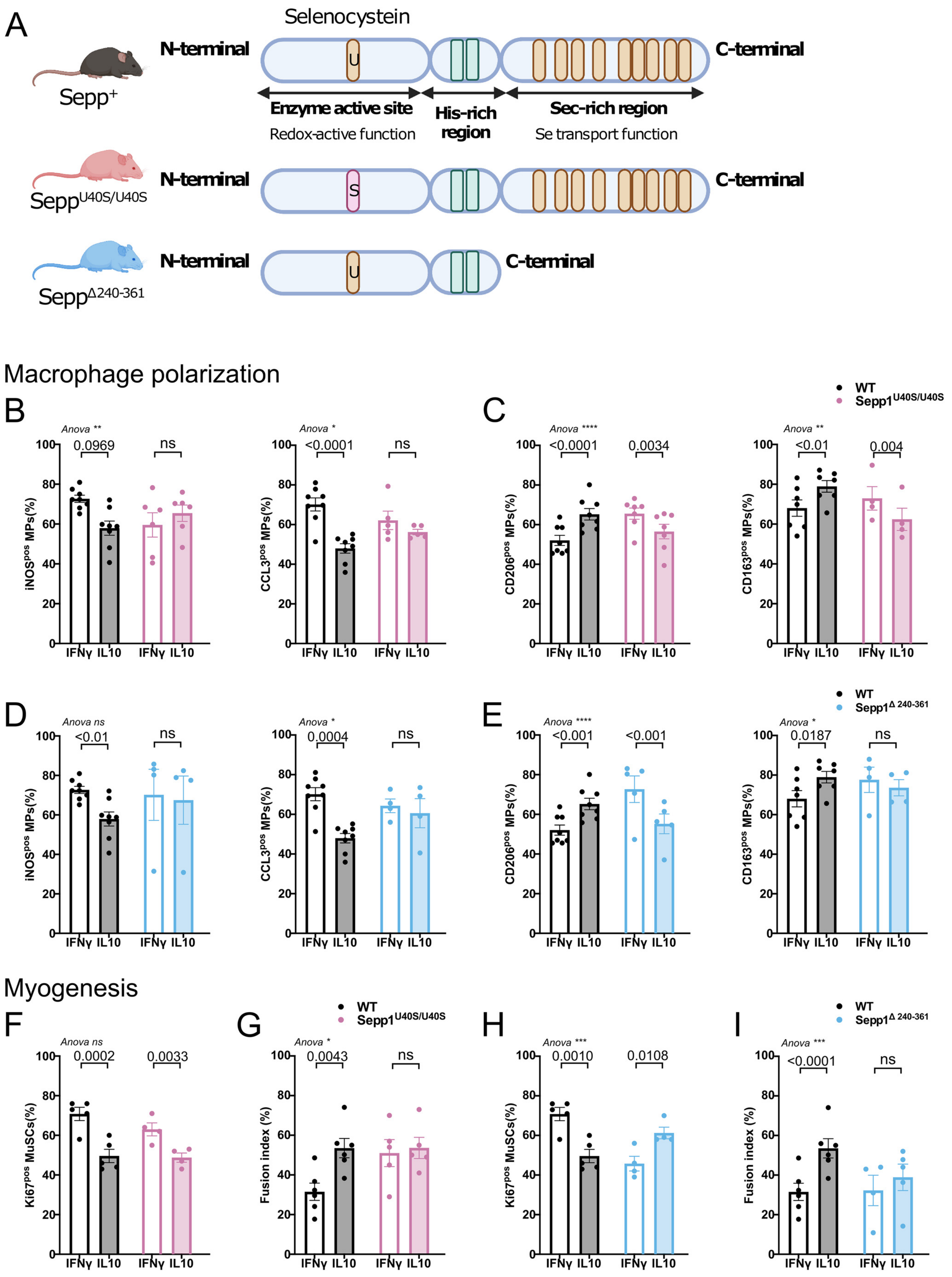
A
Selenocystein
N-terminal
C-terminal
U
Sepp+
Enzyme active site
Redox-active function
His-rich region
Sec-rich region
Se transport function
SeppU40S/U40S
S
SeppΔ240-361
Macrophage polarization
B
C
D
E
WT
Sepp1U40S/U40S
Sepp1Δ 240-361
Anova **
Anova *
Anova ****
Anova ns
0.0969
ns
<0.0001
0.0034
<0.01
0.004
0.0004
<0.001
0.0187
iNOSpos MPs(%)
CCL3pos MPs(%)
CD206pos MPs(%)
CD163pos MPs(%)
IFNγ IL10 IFNγ IL10
Myogenesis
F
G
H
I
Anova ***
0.0002
0.0033
0.0043
0.0010
0.0108
<0.0001
Ki67pos MuSCs(%)
Fusion index (%)
100
80
60
40
20
0

 © The Author(s)

Figure EV4. Effect of the loss of redox-activity and selenium transport in Sepp1 on macrophage phenotype and functions in vitro.

(**A**) Schematic representing Sepp1 structure and the mouse models having mutation impairing either the redox function ($Sepp^{U40S/U40S}$), or the selenium transport function ($Sepp^{\Delta 240\text{-}361}$). (**B–E**) Wild-type (WT), $Sepp^{U40S/U40S}$ and $Sepp^{\Delta 240\text{-}361}$ bone marrow-derived macrophages (BMDMs) were polarized into pro-inflammatory and anti-inflammatory macrophages with IFNγ and IL10, respectively and analyzed for their inflammatory status by immunofluorescence. The number of $Sepp^{U40S/U40S}$ BMDMs expressing the pro-inflammatory markers iNOS ($n = 6$–8) and CCL3 ($n = 5$–8) (**B**) and the anti-inflammatory markers CD206 ($n = 7$–8) and CD163 ($n = 4$–7) (**C**) was counted. The number of $Sepp^{\Delta 240\text{-}361}$ BMDMs expressing the pro-inflammatory markers iNOS ($n = 4$–8) and CCL3 (n = 4–8) (**D**) and the anti-inflammatory markers CD206 ($n = 5$–8) and CD163 ($n = 4$–7) (**E**) was counted. (**F–I**) WT and $Sepp^{U40S/U40S}$ BMDMs were polarized as above and conditioned medium was collected and transferred onto Muscle Stem cells (MuSCs) to evaluate their proliferation (**F**) ($n = 4$–5) and their myogenesis (**G**) ($n = 5$–6). (**F–I**) WT and $Sepp^{\Delta 240\text{-}361}$ BMDMs were polarized as above and conditioned medium was collected and transferred onto MuSC) to evaluate their proliferation (**H**) ($n = 4$–5) and their myogenesis (**I**) ($n = 5$–6). Data information: Values are given as mean ± SEM. Each dot represents one experiment using primary cells issued from one animal. Two-way ANOVA test was performed followed by multiple comparisons using Šidák test. Result of the two-way ANOVA test is shown for each graph as *$P < 0.05$; **$P < 0.01$; ***$P < 0.001$; ****$P < 0.0001$.

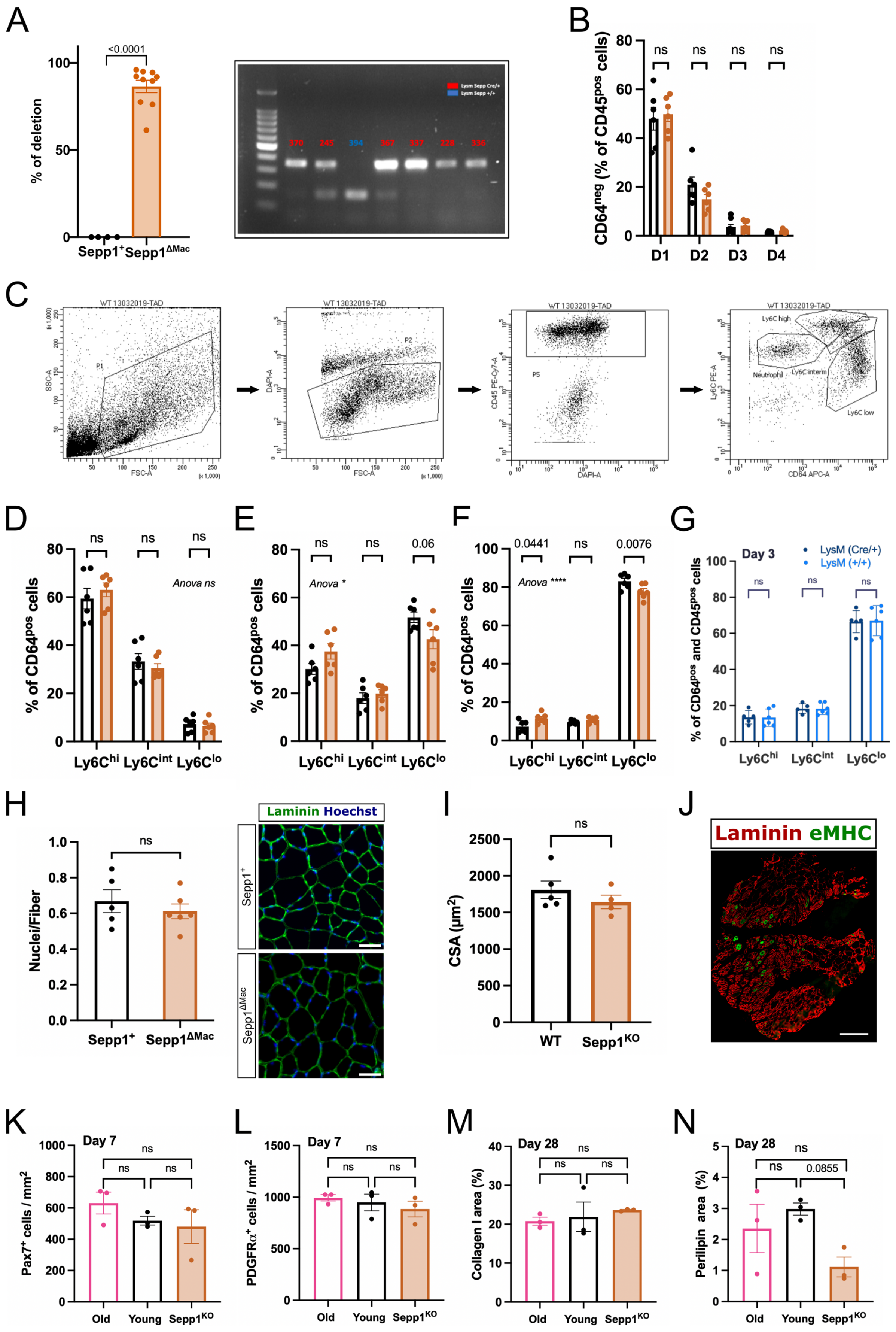
A
% of deletion
<0.0001
Sepp1+ Sepp1ΔMac
Lysm Sepp Cre/+
Lysm Sepp +/+
B
CD64neg (% of CD45pos cells)
ns
D1 D2 D3 D4
C
WT 13032019-TAD
P1
SSC-A
FSC-A
DAPI-A
P2
CD45 PE-Cy7-A
P5
Ly6C PE-A
Ly6C high
Neutrophil
Ly6C interm
Ly6C low
CD64 APC-A
D
% of CD64pos cells
Anova ns
Ly6Chi Ly6Cint Ly6Clo
E
Anova *
0.06
F
0.0441
0.0076
Anova ****
G
Day 3
LysM (Cre/+)
LysM (+/+)
% of CD64pos and CD45pos cells
H
Nuclei/Fiber
Laminin Hoechst
Sepp1+
Sepp1ΔMac
I
CSA (µm2)
WT Sepp1KO
J
Laminin eMHC
K
Day 7
Pax7+ cells / mm2
Old Young Sepp1KO
L
PDGFRα+ cells / mm2
M
Day 28
Collagen I area (%)
N
Perilipin area (%)
0.0855

Figure EV5. Effect of the loss of Sepp1 in macrophages on skeletal muscle regeneration in vivo.

(**A**) Evaluation of the depletion of Sepp1 gene in CD11b^{pos} bone marrow cells of Sepp1$^{\Delta Mac}$ mice (*n* = 4–9). Student *t* test was performed. (**B–F**) *Tibialis Anterior* (TA) muscles from Wild-type (WT) and Sepp1$^{\Delta Mac}$ mice were injected with cardiotoxin and were harvested 1, 2, 3, 4 days after the injury. (**B**) The number of CD45^{pos} CD64^{neg} cells (neutrophils) was quantified by flow cytometry as a percentage of total CD45^{pos} immune cells (*n* = 6–8). Two-way ANOVA test was performed followed by multiple comparisons using Šidák test. (**C**) Gating strategy for the analysis of macrophage subsets by flow cytometry. (**D–F**) The number of Ly6C^{pos}, Ly6C^{int} and Ly6C^{neg} macrophages was quantified by flow cytometry at day 1 (**D**), 2 (**E**) and 4 (**F**) as a percentage of total CD64^{pos} macrophages (*n* = 6). Two-way ANOVA test was performed followed by multiple comparisons using Šidák test. (**G**) LysM$^{Cre+/+}$ and control (LysM$^{+/+}$) mice were analyzed for the populations of macrophages at day 3 after injury (*n* = 5–6). Two-way ANOVA test was performed followed by multiple comparisons using Šidák test. (**H**) Uninjured WT and Sepp1$^{\Delta Mac}$ TA muscles were analyzed for the number of nuclei *per* myofiber (*n* = 5–6). Student *T* test was performed. Bars = 40 µm. (**I**) Injured WT and Sepp1$^{\Delta Mac}$ TA muscles were analyzed for the size of the regenerating myofibers (CSA) 28 days after injury (*n* = 4–5). Student *T* test was performed. (**J**) View of a total muscle section of an old mouse transplanted with young bone marrow, 7 days post injury, embryonic myosin heavy chain (eMHC) is labeled in green (a part of that picture is shown in Fig. 5F, middle panel). Bar = 500 µm. (**K–N**) Old WT mice were irradiated and bone marrow transplanted with bone marrow from either young, old or Sepp1$^{\Delta Mac}$ mice and TA muscles were injected with cardiotoxin one month later and were harvested 7 and 28 days after the injury (*n* = 3). The number of Pax7^{pos} (**K**) and PDGFRα (**L**) was counted at day 7; the area covered by collagen I (**M**) and perilipin (**N**) was quantified at day 28 after injury. One-way ANOVA test was performed followed by multiple comparisons using Tukey test. Data information: Values are given as mean ± SEM. Each dot represents one mouse. Result of the two-way ANOVA test is shown for each graph as *P < 0.05; ****P < 0.0001.
